# A heterophil/lymphocyte-selected population reveals the phosphatase PTPRJ is associated with immune defense in chickens

**DOI:** 10.1038/s42003-023-04559-x

**Published:** 2023-02-18

**Authors:** Jie Wang, Jin Zhang, Qiao Wang, Qi Zhang, Mamadou Thiam, Bo Zhu, Fan Ying, Mohamed Shafey Elsharkawy, Maiqing Zheng, Jie Wen, Qinghe Li, Guiping Zhao

**Affiliations:** 1 Institute of Animal Sciences, Chinese Academy of Agricultural Sciences, State Key Laboratory of Animal Nutrition, Beijing, 100193 China; 2 Poultry Institute, Shandong Academy of Agricultural Sciences, Ji’nan, 250100 P. R. China

**Keywords:** Animal breeding, Genetic variation, Immunogenetics

## Abstract

Quantification of leukocyte profiles is among the simplest measures of animal immune function. However, the relationship between H/L ratio and innate immunity and the measure’s utility as an index for heterophil function remains to be analyzed. Variants associated with H/L ratio were fine-mapped based on the resequencing of 249 chickens of different generations and an F2 segregating population generated by crossing selection and control lines. H/L ratio in the selection line was associated with a selective sweep of mutations in protein tyrosine phosphatase, receptor type J (*PTPRJ*), which affects proliferation and differentiation of heterophils through its downstream regulatory genes. The SNP downstream of *PTPRJ* (rs736799474) have a universal effect on H/L, with CC homozygotes exhibiting improved heterophil function because of downregulated *PTPRJ* expression. In short, we systematically elucidated the genetic basis of the change in heterophil function resulting from H/L selection by identifying the regulatory gene (*PTPRJ*) and causative SNP.

## Abstract (web summary)

Genomic and transcriptomic profiling in Jingxing yellow chickens selected for a heterophil-lymphocyte ratio (H/L) provides further insight on the genetic basis of H/L and identifies PTPRJ as a relevant factor.

## Introduction

It is now well known that different species have evolved their immune function to adjust to their specific environmental and ecological contexts^1^. In general, species that are exposed to a greater diversity of pathogens and suffer greater risk of disease should have developed stronger immune defense^2^. Quantification of leukocyte profiles is one of the simplest measures of animal immune function^3^, and has in recent decades become an increasingly popular and widely applied tool in the fields of ecology, ecophysiology, and conservation physiology^4^. In birds, intrapopulation variation in the heterophil to lymphocyte (H/L) ratio has been reported to reflect a broad spectrum of stressors, with ample evidence indicating that H/L ratio increases due to parasitic infestation and certain infections^5,6^. Although changes in an individual’s H/L ratio can occur rapidly (in tens of minutes) in response to acute stressors^7^, it has been shown that the reference H/L ratio measured under normal physiological function (without acute stress) maintains a very consistent level from a long-term perspective (trans-seasonal)^8^. In terms of immune function, birds with initially low H/L ratios have stronger antibody responses to *Brucella abortus* than those with high H/L ratios^9^. Likewise, after infection with *Salmonella typhimurium* (ST), chickens with low H/L exceeded those with high H/L in terms of all studied immune response indicators including antibody titer, cellular immunity, phagocytic activity, cortisol concentration, bursa, and body weight^10^. Moreover, H/L ratios are linked to basic fitness components, with high H/L ratio predicting lower recruitment and survival in some passerine populations^6,11^. Overall, studies have shown that the H/L ratio is an important feature of physiological evolution in birds, and one indicative of an interplay between immunity, physiology, and ecology^12^.

Lymphocytes and neutrophils/heterophils are the two most abundant types of white blood cells, normally comprising up to 80-90% of all leukocytes^4^. Neutrophils/heterophils form the first line of innate cellular defense against pathogens and actively participate in inflammatory lesions^13–15^. Specifically, infection by pathogenic microorganisms results in acute inflammation, heterophils interact with vascular endothelial cells at the receptor level and migrate to the inflammation site^16^, where to protect the host from damage they kill pathogens through trapping, phagocytosis, oxidation, degranulation, and other processes^14,17^. Avian heterophils lack peroxidase and hence is deficient in producing large amounts of hydrogen peroxide and superoxide anion, yet they still possess potent bactericidal ability^18^. Research on the non-oxygen-dependent bactericidal effects of heterophils has mainly focused on antimicrobial peptides^15^, β-defensins, cathepsins, lysozyme, β-glucuronidase, and α-glucosidase^13^. Notably, for independent individual chicken, the phagocytosis and pathogen-killing capability of heterophils relates to breed and cross^14,19,20^, and chicken populations with stronger heterophil function have stronger disease resistance against pathogens such as *Salmonella enteritidis*^21^, *Enterococcus gallinarum*^22^, and *Eimeria tenella*^23^.

**Fig. 1:**
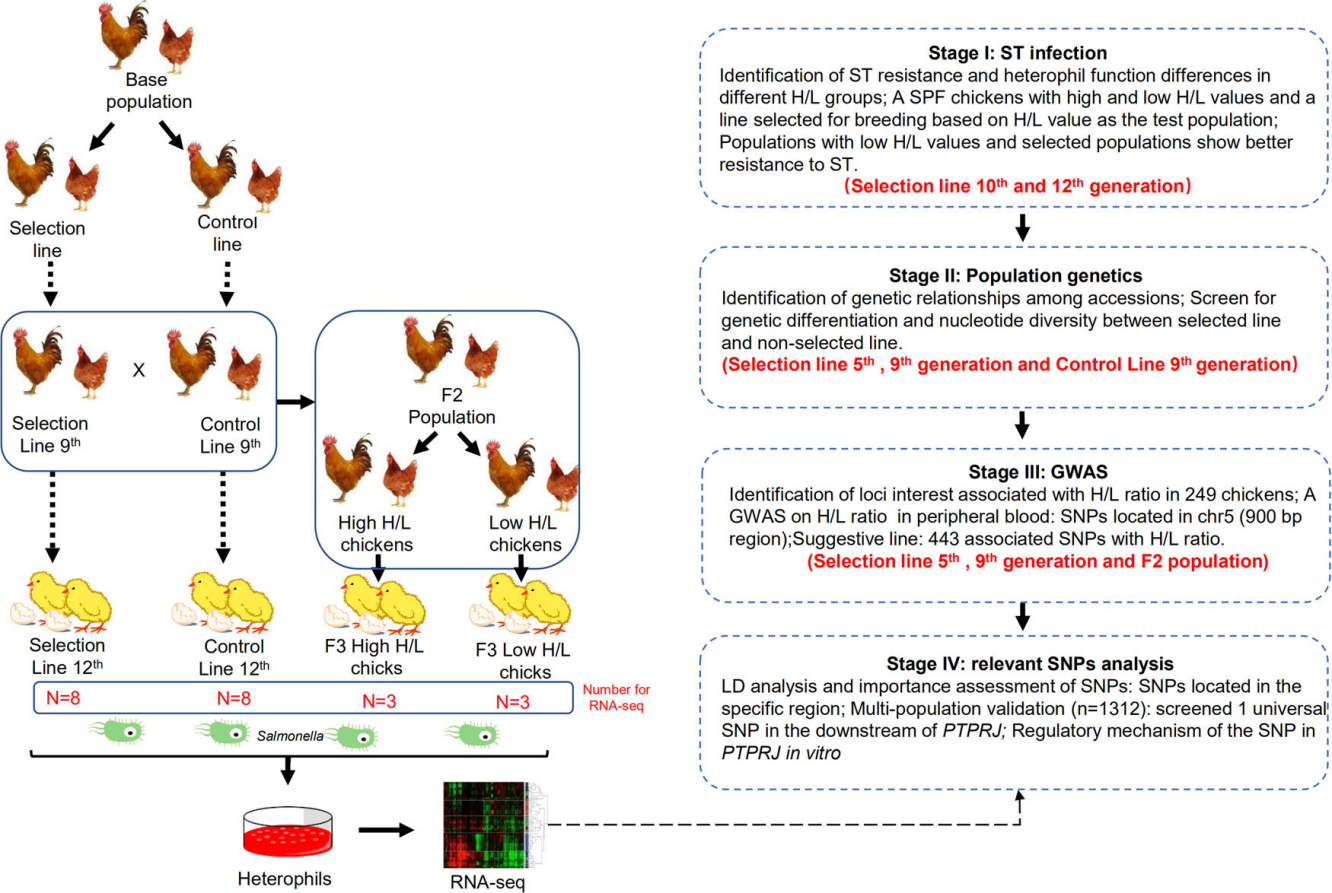
Graphic representation of study design. ST *Salmonella* typhimurium, H/L Heterophil to lymphocyte ratio, GWAS Genome-wide association study, SNPs Single nucleotide polymorphisms, LD Linkage disequilibrium (LD), *PTPRJ* Protein tyrosine phosphatase, receptor type J.

The aim of this study was to reconstruct the evolutionary history of H/L ratio (using chickens as a model) and to examine how the genome and heterophil function correlates in birds. We hypothesized that the evolution of H/L ratio in birds should be primarily shaped by stabilizing selection, and that chickens with lower ratios should have stronger resistance to diverse pathogens. To test the hypothesis, we used a line of Jingxing yellow chickens (JXH) with selection on heterophil to lymphocyte ratio for ten generations. We profiled the genomes of chickens from the selection and corresponding control lines to reveal associated selection signatures, and performed a *Salmonella* challenge and calculated the mortality difference between selected and non-selected lines. We also carried out fine mapping of phenotypes based on the resequencing of chickens of different generations and an F2 segregating population generated by crossing of the selection and control lines (Fig. 1, Supplementary Fig. 1).

## Results

### Populations with different H/L ratios show different degrees of *Salmonella typhimurium* (ST) resistance

The insufficient immunity of chicks makes them susceptible to a variety of invading pathogens, such that infection is one of the most common causes of chick death. In this study, we determined resistance to *Salmonella* among 319 chicks (Supplementary Data 1). We selected specific-pathogen-free (SPF) White Leghorn chickens and Chinese native chickens (Jingxing yellow chickens, JXH), grouped them according to H/L, and infected them with *Salmonella*. The results showed that chicks with low H/L had lower mortality. To determine whether H/L-associated resistance or susceptibility to *Salmonella* was heritable, we built a resource population by crossing parents with high or low H/L, from which distinct families were generated. We found that the chicks from low H/L parents also had lower mortality than those having high H/L parents (*P* < 0.05) (Table 1).

**Table 1:** Mortality rate of chicks by group after *Salmonella typhimurium* (ST) infection.

Breeds	Group	Mortality rate (deadline)	Number	Strain
Selection line 10th generation Batch 1	selected	2.8% (72 hpi)	35	ST:21484, CICC
	non-selected	11.6% (72 hpi)	60	
Selection line 10th generation Batch 2	selected	12.0% (72 hpi)	116	ST:21484, CICC
	non-selected	20.0% (72 hpi)	30	
Selection line 12th generation	selected	4.44% (24 hpi)	90	ST:21484, CICC
	non-selected	10.00% (24 hpi)	90	

*hpi* hours postinfection.

### Selection for H/L to increase *Salmonella* resistance and cause genetic differentiation

**Fig. 2:**
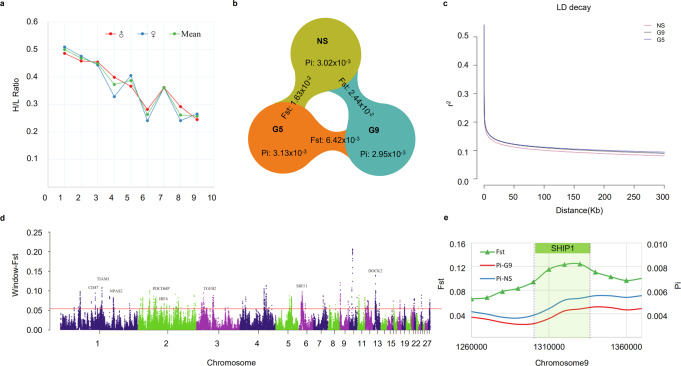
Selection for H/L causes genetic differentiation. **a** Phenotype progress over the course of H/L breeding. **b** Summary of nucleotide diversity (Pi) and population divergence (Fst) across selected and non-selected groups. Values in each circle represent nucleotide diversity for the group, and values between pairs indicate population divergence (Fst). NS, non-selection line; G5, 5th generation; G9, 9th generation. **c** Decay of linkage disequilibrium (LD) in the three groups, measured by r2. **d** Pairwise fixation index (Fst) in 40-kb sliding windows across autosomes between G9 and NS. The dashed horizontal line indicates the Fst cutoff (top 1%). Genes located in divergence regions annotated by Ensembl BioMart are indicated by their gene names. **e** CDRs are enriched for sites on chromosome 9, the gene set for the FcR and ITIM pathways.

Disease resistance in chickens can be improved by selection for immune ability. In particular, H/L is a trait that can directly represent disease resistance at the cellular level and thus can be used as an index to measure *Salmonella* resistance. We compared the H/L of generations 1 through 9, and found that the H/L of the selected line was reduced from 0.5 to about 0.25 (Fig. 2a). Using the entire dataset, the pedigree-based BLUP model revealed the heritability of the H/L ratio to be 0.13.

To understand the genetic changes that occurred during the selection process, we selected 227 individuals for whole-genome re-sequencing and called SNPs across the genome. In JXH accessions, overall nucleotide diversity as measured by the Pi value was 3.03 × 10^−3^, with the non-selected population showing higher diversity (3.02 × 10^−3^) than the 9th generation of the selection line (2.95 × 10^−3^); this suggests that genome-wide polymorphism was reduced after selection (Fst = 2.44 × 10^−2^) (Fig. 2b and c, Supplementary Data 2, Supplementary Figs. 2 and 3). Annotation of candidate divergent regions (CDRs) identified a series of genes relating to inflammation, the intestinal barrier, cell migration, microbial defense, and other immune-related functions (*ADAMTS5, CLDN8, TIAM1, SOD1, TJP1, MYO1E, EIF4G3, THBS2, VSIG4, HSF3, CORIN, ST6GALNAC3*, and *SHIP1*) (Fig. 2d and e, Supplementary Fig. 4); details of these genes and their respective expression in immune organs are given in the supplementary materials (Supplementary Data 5, Supplementary Fig. 5). Notably, the TIAM1-RAC1-NOX2 signaling axis affects the production of reactive oxygen species (ROS)^24^, as phagocyte-like NADPH oxidase-2 (NOX2) is their predominant cytosolic source. After ST infection, *TIAM1* expression in the selected line was significantly up-regulated in the spleen and cecal tonsils, which are rich in heterophils and macrophages. Another gene of interest is *SHIP-1* (*INPP5D*), the protein product of which has roles in a variety of immune-related functions: it acts as a negative regulator of B-cell antigen receptor signaling; mediates signaling from the F_C_-gamma-RIIB receptor (FCGR2B) and so plays a central role in terminating signal transduction from receptor systems that activate immune/hematopoietic cells; negatively regulates myeloid cell proliferation/survival, chemotaxis and mast cell degranulation; regulates proliferation of osteoclast precursors; and is involved in the control of cell-cell junctions, in CD32a signaling in neutrophils, and in the modulation of EGF that induces phospholipase C activity^25^.

In addition to the abovementioned genetic changes, we found that populations continuously selected for low H/L had increased *Salmonella* resistance (Table 1). Phagocytosis, which is enacted by heterophils in avian, is a key element of host innate immunity against invading microbial pathogens. Upon detection of pathogens through a network of intracellular signaling pathways and the release of and response to cytokines and chemokines, heterophils employ a repertoire of microbial killing mechanisms, including production of an oxidative burst and cellular degranulation^13,26^. Two genes related to heterophil function (*TIAM1* and *SHIP-1*) were in the set selected during the breeding process, which may explain the observed increase in *Salmonella* resistance. In order to prove that these are true signatures of selection, we developed a statistical test to show that the effects of random drift are negligible. The results are illustrated in Supplementary Fig. 6; in short, the simulation based on the NS population shows that the changes in allele frequency caused by genetic drift over the nine generations are smaller than those caused by H/L selection.

### Identification of genes under selection in H/L selected chickens

**Fig. 3:**
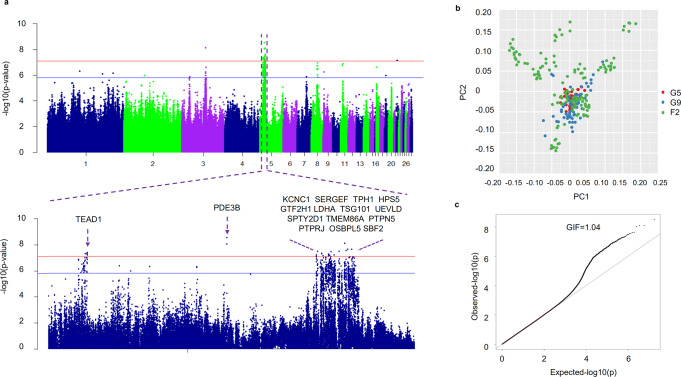
Selective sweep associated with lower H/L ratio. **a** A GWAS on H/L in chicken peripheral blood identified a 1-Mb region on chromosome 5. Red horizontal dashed lines indicate the Bonferroni-corrected significance threshold (7.1 × 10^−8^). **b** Population structure evaluated on the basis of the first two principal components. **c** Quantile–quantile (Q–Q) plot of the GWAS results. The x-axis shows the expected *p* values under the null hypothesis, and the y-axis the observed *p*-values. The GIF (genomic inflation factor) was 1.04 for H/L, which suggests that the population structure was well controlled.

To investigate genetic regulation of H/L in chickens, we performed a genome-wide association study (GWAS) examining a total of 8,788,385 single nucleotide polymorphisms (SNPs) in 249 chickens. *P* values were corrected with a strict Bonferroni adjustment based on LD pruning^27^, in which the number of independent statistical comparisons was defined as the sum of the independent LD blocks plus singleton markers^28^. Ultimately, 640,054 independent SNPs were used to determine the *P* value thresholds for genome-wide significance (7.81 × 10^−8^) and suggestive association (1.56 × 10^−6^). A strong association signal was detected in a 1-Mb region of chromosome 5 (chr5: 12,400,000–13,300,000) (Fig. 3, Supplementary Data 6, 7), and related genes showed differential expression in different immune organs (Supplementary Fig. 5); these genes included protein tyrosine phosphatase, receptor type J (*PTPRJ*).

**Fig. 4:**
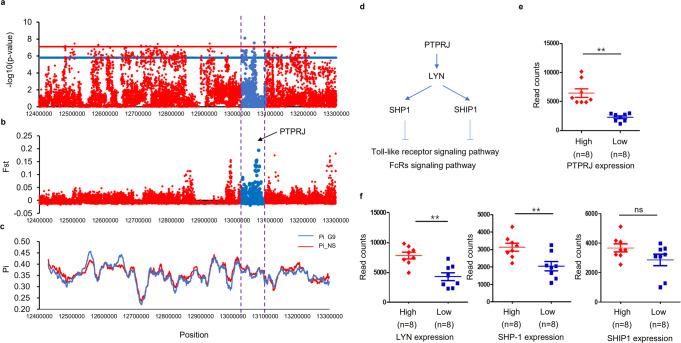
Functional genomic basis of H/L values in chickens. **a** GWAS revealed strong association of chicken H/L with a 1-Mb region on chromosome 5. **b** Fixation index (Fst) between G9 and NS chickens for all SNPs along the 1-Mb region. **c** Nucleotide diversity (Pi) of G9 (blue line) and NS chickens (red line) from 12.4 to 13.3 Mb on chromosome 5. **d** Schematic overview of *PTPRJ* function. **e** mRNA expression of *PTPRJ* by RNA-seq in individuals having high and low *PTPRJ* expression. **f** mRNA expression of *LYN*, *SHP-1*, and *SHIP-1* by RNA-seq in individuals having high and low *PTPRJ* expression. The indicated significance (**) is based on one-way ANOVA, ns: not significant (*p* > 0.05). Data are expressed as the mean ± standard deviation (SD).

**Fig. 5:**
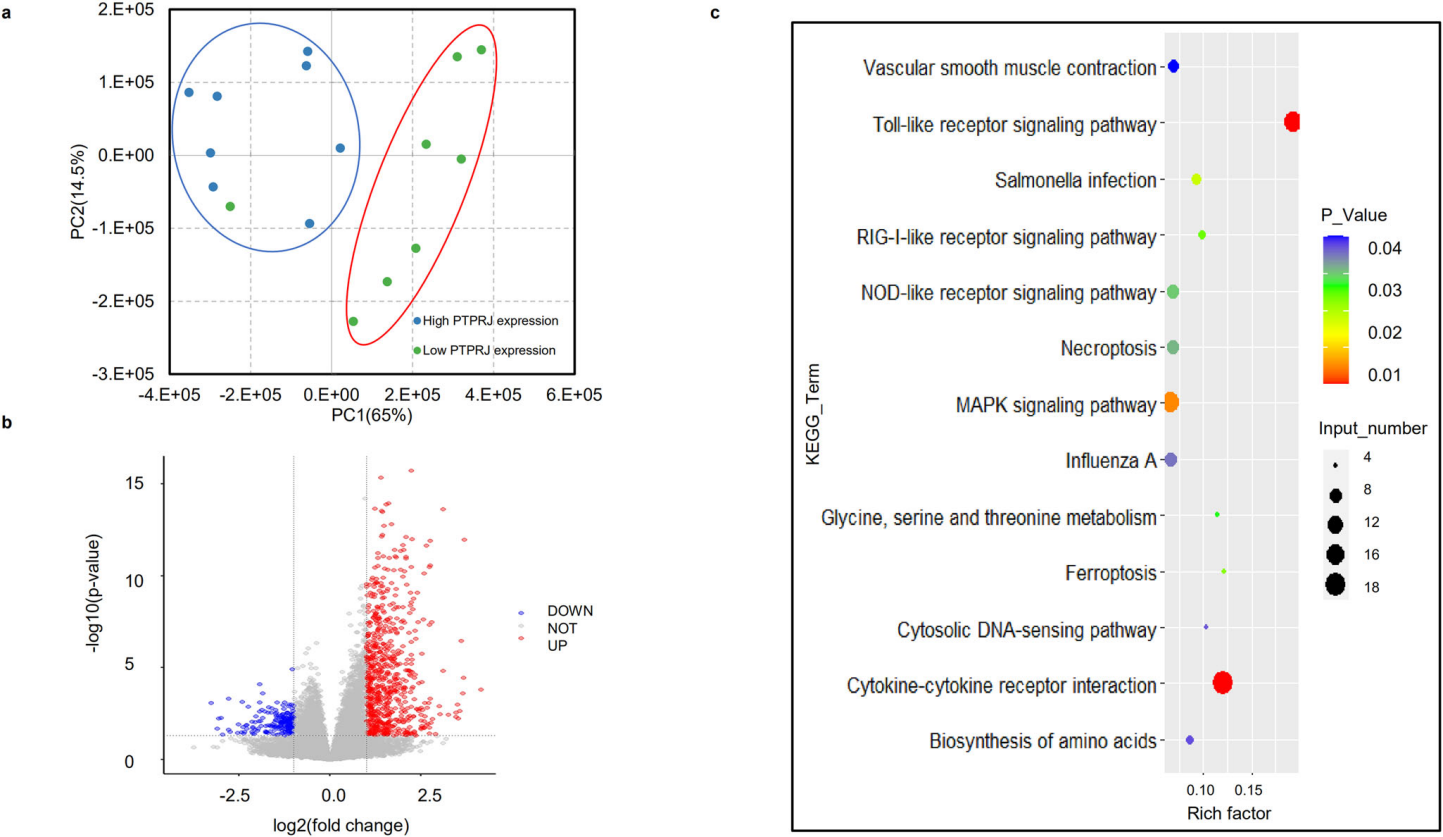
Gene expression profiles of heterophils from individuals grouped by *PTPRJ* expression level. **a** RNA-seq analysis and identification of genes differentially expressed between different groups of chicks. PCA was performed on the RNA-seq data for all genes identified in heterophils. **b** Volcano plot showing DEGs. Red dots represent significantly upregulated genes (|log2 FC| ≥ 1 and *P* < 0.05); blue dots represent significantly downregulated genes; and gray dots represent genes with no significant change. **c** Pathways enriched in DEGs. The y axis represents all significantly enriched pathway terms in DEGs. The x axis represents rich factors as defined by the degree of DEG enrichment (number of DEGs in term/number of all genes in term). Color represents significance, and bubble size represents the number of DEGs in the pathway.

The combination of GWAS and selective sweep analysis is an excellent strategy for finding key genes related to desired traits. As such, we focused here on screening for candidate genes and universal SNPs involved in regulating H/L using the GWAS and selection signature data. After multiple generations of breeding, H/L values continued to decrease (Fig. 2a), and further analysis identified a significant selection signature in the associated chromosome 5 region in terms of both nucleotide diversity (Pi) and population divergence (Fst, top 1%), suggesting that *PTPRJ* in particular underwent selection in the selected line (Fig. 4a–c, Supplementary Data 8). Studies have shown that neutrophils from *Ptprj*^−/−^ mice have stronger chemotaxis and phagocytosis, and that *PTPRJ* exerts a negative regulatory effect through the *LYN* gene (Fig. 4d)^29^, which plays an important role in modulation of neutrophil granulopoiesis, apoptosis, and adhesion^30^. We found that heterophil expression of *PTPRJ* and its downstream regulatory genes differed between low H/L (n = 3) and high H/L (n = 3) individuals in the F3 population (Supplementary Fig. 7). We further grouped individuals by *PTPRJ* expression level (high expression, n = 8; low expression, n = 8), and measured the expression of downstream genes, confirming that *PTPRJ* expression was positively correlated with that of downstream regulatory genes (Fig. 4e and f). Meanwhile, gene expression profiling and comparison of profiles grouped by *PTPRJ* expression level (Fig. 5a and b) identified a total of 938 DEGs (|log2 FC| ≥ 1, with *P* < 0.05) in heterophils, of which 204 were downregulated and 734 upregulated. Pathway analysis of these DEGs revealed several significantly enriched pathways (*P* < 0.05) (Fig. 5c). As expected, these included some *Salmonella*-related pathways, e.g., the Toll-like receptor signaling pathway, *Salmonella* infection signaling pathway, MAPK signaling pathway, and cytokine interaction pathway. The GO analysis results are given in Supplementary Data 11, among which significantly enriched (*P* < 0.05) terms mainly related to the innate immune response, Myd88-TLR receptor signaling, neutrophil chemotaxis, respiratory burst, and related cytokine production. The above GO enrichment results show that individuals with differential expression of *PTPRJ* also have heterophils in their blood undertake different functions after *Salmonella* infection. The differential expression analysis based on *PTPRJ* expression also yielded the differential expression of its downstream genes, as shown in Fig. 4e and f. Among those genes, *LYN* and *SHP1* exhibited expression levels consistent with the H/L high and low group data; their expression trends were also consistent with that of *PTPRJ*, and the differences achieved significance. We speculated that individuals with low H/L have lower expression of *PTPRJ*, leading to down-regulation of the immunosuppressive genes downstream (Supplementary Fig. 8).

### Function and fate of mutations for SNPs in *PTPRJ*

**Fig. 6:**
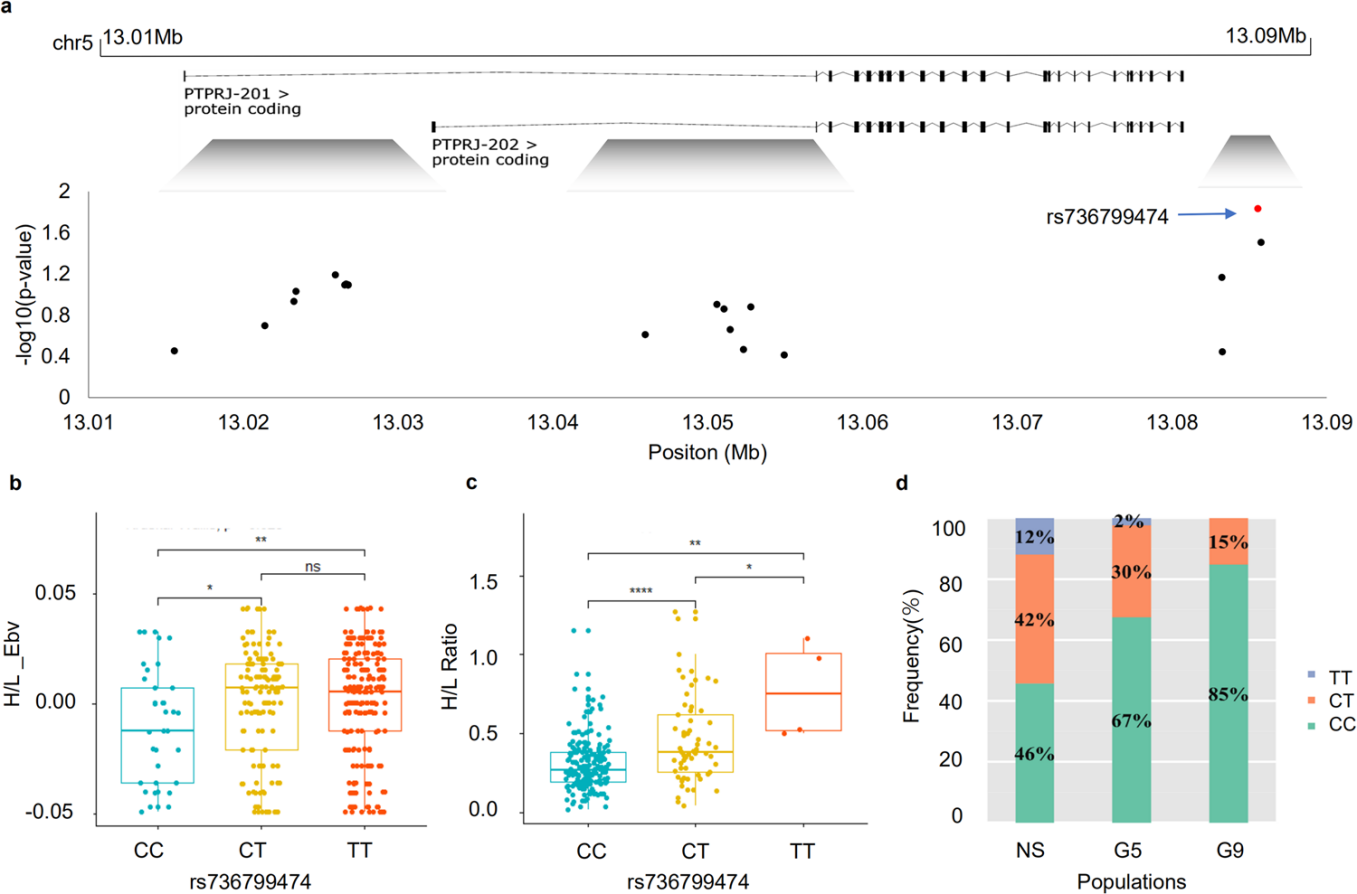
Identification of the universal SNP associated with chicken H/L (rs736799474). **a** and **b** Effects of rs736799474 alleles in JLH chickens. Genome annotation according to chicken reference genome (ftp://ftp.ensembl.org/pub/release-96/fasta/gallus_gallus/dna/). **c** Effects of rs736799474 alleles in the H/L selection population. **d** Allele distributions in the selection line and non-selection JXH chickens. Middle line represents the median, upper and lower limits of box represent the upper and lower quartiles in bar plot.

We genotyped *PTPRJ* by MassARRAY technology 384 individuals from another Chinese native chicken breed (Jin Ling Hua, JLH), then performed a multi-population validation analysis of the relationship between genotype and H/L-estimated breeding value (EBV) in 1199 JLH chickens (Fig. 6a). Consistent with the H/L selected line, we found a significant effect of rs736799474 on H/L in the tested populations (Fig. 6a and b), but no significant effect for the other SNPs examined. Accordingly, rs736799474 was considered to be a universal SNP associated with H/L in chicken peripheral blood. Individuals with the CC genotype exhibited significantly lower H/L than those with the TT genotype (Fig. 6c), suggesting that the C allele of rs736799474 makes an important contribution to H/L in chickens. We also tracked changes in the allele frequency of rs736799474 during H/L selection. As breeding generation increased, the frequency of the CC genotype gradually increased, while the TT genotype gradually disappeared (Fig. 6d). In particular, we simulated the frequency of the SNP rs73679947 in the *PTPRJ* gene. The frequency of the C allele in the NS population was 0.67, which was taken as the initial value; after nine generations, the frequency was about 0.51-0.79. In contrast, the frequency in the G9 population was 0.92, significantly higher than the simulated value.

**Fig. 7:**
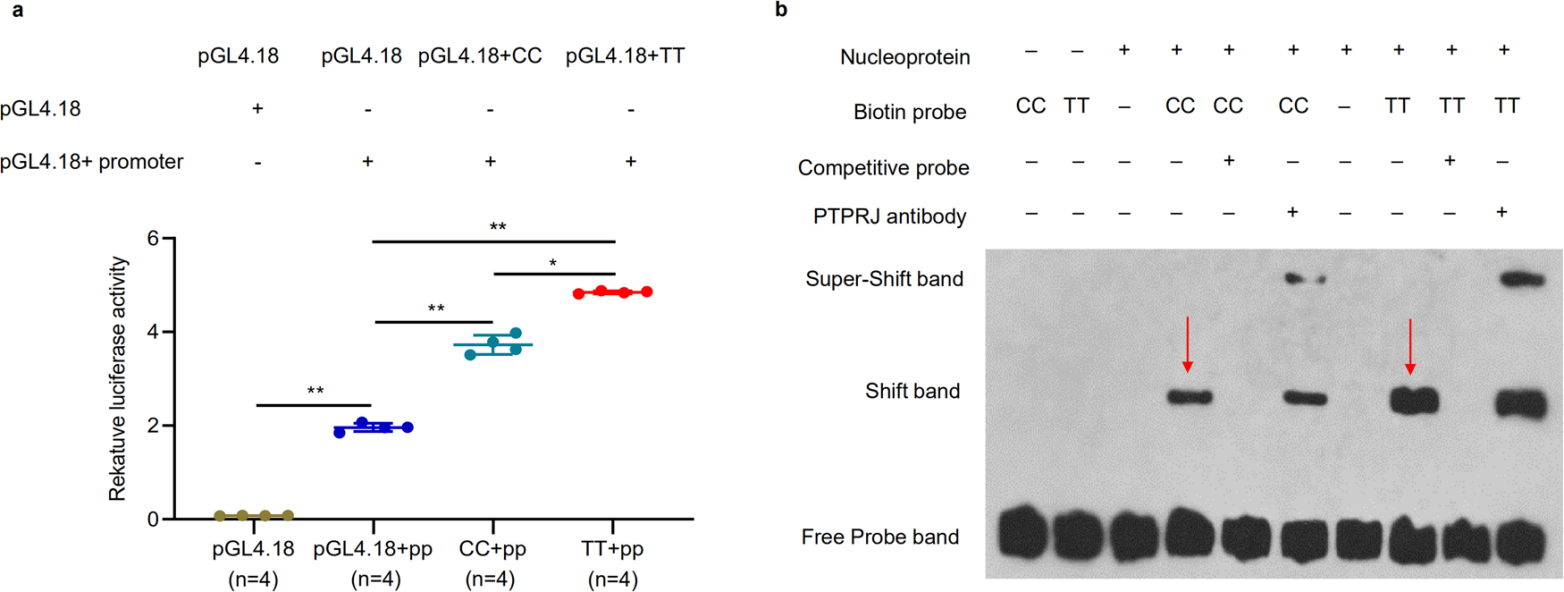
Effects of causative rs736799474 on enhancer activity and binding of heterophil nuclear proteins. **a** Dual-luciferase reporter assay concerning the effect of the rs736799474 [T] to [C] transition on activity of a *PTPRJ* enhancer. Fragments containing the candidate variations were synthesized: Fragment 1 containing allele C, and Fragment 2 containing allele T. Each fragment was cloned into the 5’ end of a pGL4.18 vector for analysis of enhancer activity (enhancer-D). Empty pGL4.18 (Basic) was used as a negative control, and pGL4.18 containing the *PTPRJ* promoter (*PTPRJ* Promoter) was used as a reference for enhancer activity. Data represent the mean ± SD from three biological replicates per vector. Activities of the pGL4.18 + C and pGL4.18 + T vectors were compared using Student’s *t*-test. ** indicates *P* < 0.01, * indicates *P* < 0.05. **b** Effects of rs736799474 alleles on binding of heterophil nuclear proteins. EMSA was performed using biotin-labeled oligo probes containing either rs736799474 allele (C) or allele (T). Two microliters of nuclear proteins (5 μg/μl) from heterophils were incubated with 40 pmol of biotin-labeled probes. Specific binding was confirmed using 200-fold excess unlabeled cold probes containing the appropriate rs736799474 allele (C cold probe or T cold probe). Arrows indicate changes in protein binding capacity between alleles.

We additionally performed dual-luciferase reporter assays to explore how the downstream rs736799474 SNP regulates *PTPRJ* expression. To investigate the effect of the rs736799474 [T] to [C] transition on *PTPRJ* transcription activity, we generated for each allele a *PTPRJ* promoter-reporter plasmid consisting of a 600 bp fragment in the pGL4.18 vector (Supplementary Fig. 9). Separate transfection of these plasmids into chicken fibroblast DF_1_ cells resulted in significantly different activities, with the C allele showing lower activity relative to the T allele (Fig. 7a). Thus, the rs736799474 [C] allele weakens the effect of the enhancer located downstream of *PTPRJ*. Electrophoretic mobility shift assays (EMSAs) further revealed differences in nuclear protein binding capacity between TT and CC genotypes (Fig. 7b, Supplementary Fig. 10). Taken together, these results led us to speculate that the SNP rs736799474 is the causative mutation responsible for the observed differences in *PTPRJ* expression, and that it may induce differential expression of the gene by altering protein binding.

## Discussion

The results of this study provide compelling evidence for the evolution of H/L ratio as an important physiological adaptation in birds. First, we detected a relatively strong selection signal in the process of H/L ratio selection, suggesting that stabilizing selection is the force responsible for the evolution of H/L ratio in birds. Second, we observed strong support for correlated evolution of avian H/L ratio with resistance to diverse pathogens. We evaluated chicken populations having different H/L ratios in terms of their resistance to *Salmonella*. Our findings suggest that selection for low H/L could enhance resistance to *Salmonella* by enhancing heterophil function. Finally, we also combined data from the selection signal, GWAS, and RNA-seq, and identified the gene *PTPRJ* as associated with H/L and heterophil function.

In chickens, variability in the expression and function of heterophil-related genes is associated with pathogen resistance^14^. Resistance to *Salmonella* is of particular interest as this zoonotic pathogen is transmitted through consumption of contaminated eggs and a major cause of food-borne illness in humans; *Salmonella enteritidis* (SE) accounts for more than three-quarters of cases of food-borne salmonellosis^9^, and chicken consumption is a major risk factor in SE infections^10^. In chickens, the inflammatory reaction induced by ST or SE often limits infection to the gastrointestinal tract, where it can establish a carrier state and become a potential source of contamination in poultry products; that is, birds with subclinical salmonellosis can persist undetected in production flocks and transmit zoonotic bacteria to the food chain^31^. Chicks infected with *Salmonella* after hatching can likewise be persistently colonized, and the bacteria can infect eggs laid by adult chickens^32^; furthermore, reduced growth and reproductive performance may occur as a result of microbial infection^33^. Genetically enhanced resistance through augmentation of the immune response is an alternative to the use of antibiotics in maintaining healthy food-producing animals, specifically improving chicken resistance to the *Salmonella* carrier state and to salmonellosis^34,35^. The reported heritability of chick survival after *Salmonella* challenge ranges from 0.14 to 0.62^36^, and that of the number of bacteria in internal and immune organs from 0.02 to 0.53^37–40^. Genomic regions associated with resistance to *Salmonella* challenge have been identified based on bacterial burden in multiple chicken populations^40–42^. In addition, selection on heterophil expression profile has been proposed as a method of increasing *S. enteritidis* resistance^43^. Previous studies have shown that, after ST infection, chickens with low H/L out-perform those with high H/L in all studied immune response variables, including antibody titer, cellular immunity, phagocytic activity, cortisol concentration, bursa, and body weight^44^. In this study, we compared populations having different H/L ratios in terms of mortality after ST infection, and found the low H/L population to exhibit better *Salmonella* resistance and heterophil function. Based on this finding, we constructed a H/L selected line, achieving significant reduction of H/L value after multiple generations of selection. We then conducted *Salmonella* challenge tests on selected and nonselected populations. Compared with the non-selected line, selected individuals showed enhanced resistance to *Salmonella* and heterophil function; thus, resistance to *Salmonella* and heterophil function can both be improved by H/L breeding. This study further confirmed the connection of H/L ratio with basic fitness and immunity, which can to a certain extent explain why high H/L ratio predicts lower recruitment and survival in some passerine populations^6,11^.

Being the counterpart of mammalian neutrophils, heterophils have the function of phagocytosis and killing pathogens; they are one of the most important components in the avian host’s immune response to pathogens and an integral part of the avian innate defense^14,15^. These cells have a very short lifespan under normal physiological conditions; they differentiate from bone marrow and circulate in the blood, where they remain in an inactive state until undergoing programmed apoptosis within five days, on average. Once heterophils have been summoned to respond to an infection, they can exit the blood and enter an activated state in about half an hour, whereupon they start their phagocytic function and produce cytokines and bactericidal substances. After leaving the blood, they are affected by the chemoattractants such as complement fragment C5a (inflammatory signals that a neutrophil recognizes), bacterial protein fragment f-met peptide, and other chemotactic factors to enter the tissue and migrate to the site of inflammation. This system involves selectin ligand binding to make the heterophils roll, integrin-ICAM interactions to stop them, and chemoattractants and their receptors to facilitate heterophil exit from the blood^45^. We selected heterophils from eight high-*PTPRJ* and eight low-*PTPRJ* individuals for RNA-seq. The results showed that those having high and low *PTPRJ* expression could be distinguished according to the gene expression profiles of their heterophils, which suggested that regulatory genes downstream of *PTPRJ* may also be differentially expressed. KEGG pathway enrichment analysis of the DEGs revealed significant enrichment of the Toll-like receptor signaling pathway, *Salmonella* infection signaling pathway, MAPK signaling pathway, and cytokine interaction pathway related to *Salmonella* infection. We focused on the expression of genes involved in the MyD88-TLR signaling pathway, *Salmonella* infection, and phagocytosis, respiratory burst, chemokine production, and neutrophil chemotaxis related to heterophil function. In this study, we identify the *PTPRJ* gene and its downstream regulatory pathways as potentially affecting H/L and heterophil function. *PTPRJ* has been shown to exert a negative regulatory effect through the *LYN* gene^29,46,47^, which encodes the primary kinase responsible for phosphorylating inhibitory receptors (PIR-B, SIRPa, FcRgIIb) or cytoplasmic molecules such as DOK1, which in turn recruiting phosphatases (SHP-1 and SHIP-1) to dampen intracellular pathways. Deficiency in LYN kinase tends to result in hyperactive immune cells; for example, neutrophils from LYN-deficient mice show exaggerated adhesion and subsequent activation in response to integrin ligands (ICAM-1, fibrinogen, or fibronectin), which can be attributed to reduced recruitment and activation of SHP-1^30^. When adhered to surfaces coated with either cellular counter-receptors (ICAM-1) or extracellular matrix proteins (fibrinogen or fibronectin) that engage integrins, *Lyn*^−/−^ neutrophils display a hyper-adhesive phenotype along with enhanced respiratory burst and secondary granule release^48^. In vivo, the major mechanism for limiting neutrophil numbers is apoptosis. Extracellular stimuli such as proinflammatory cytokines, cell adhesion, and phagocytosis can modulate neutrophil apoptotic death^49^; however, *Lyn*^−/−^ neutrophils and *Ship-1*^−/−^ neutrophils do not manifest enhanced apoptosis with such stimuli^50^. Recent studies have shown that *Ship*^−/−^ mice exhibit increased G-CSF production^51^ and significantly increased granulocyte numbers^52^; likewise, chemical inhibition of SHIP-1 promotes a profound increase in circulating granulocyte numbers^53^. *Lyn*^−/−^ mice also manifest increased numbers of myeloid precursors that demonstrate enhanced sensitivity to granulocyte-macrophage colony-stimulating factor (GM-CSF), which might be secondary to impaired phosphorylation of ITIM-containing receptors^54^. LYN has also been reported to act as a negative regulator of TLR4 signaling^46,47^; namely, bone marrow-derived macrophages isolated from *Lyn*^−/−^ mice and stimulated with LPS produce more IL-6, TNF-a, and IFN-a/b than their wild-type counterparts. In agreement with those ex vivo studies, increased amounts of TNF-a, IL-6, and IFN-a/b were found in the serum of *Lyn*^−/−^ mice injected with LPS. Taken together, these results indicate that LYN is involved in the downregulation of both the MyD88- and TRIF-dependent pathways of TLR4^55^. Notably, *SHIP-1* is downstream of *PTPRJ*. In our analysis of the H/L selection line and non-selected populations, selection signals were evident for both *PTPRJ* and *SHIP-1*, suggesting that the enhancement of heterophil function associated with low H/L might be due to the actions of those two genes.

In summary, we reported a H/L-based selection experiment and examined genome and heterophil function correlates in birds. We identified the gene *PTPRJ* as a regulator of H/L, and the universal SNP rs736799474, located downstream of *PTPRJ*, as associated with decreased H/L in the peripheral blood of Chinese native chickens. The results of this study strongly suggest that H/L is indicative of an interplay between immunity, physiology, and ecology, and can be used as an adaptive trait. Our findings help to improve our understanding of the genetic basis through which selecting on H/L can improve disease resistance in chickens.

## Methods

### Ethics statement and animals

All animals and experimental protocols used in this study were approved by the Beijing Institute of Animal Science, Chinese Academy of Agricultural Sciences (the scientific research department responsible for animal welfare issues) (No.: IASCAAS-AE20140615).

In this study, experimental chickens (JXH) were selected on H/L, with the base population consisting of 200 males and 500 females. All individuals in each generation had H/L measured at 56 days of age. Individuals with low H/L were selected to build 30 families, each having a male:female ratio of 1:3; that is, 30 roosters and 90 hens were used to breed the next generation, while avoiding inbreeding. At the same time, a control population (non-selection line) was maintained using pooled semen to breed each next generation. About 700-800 individuals were hatched in each respective generation of the selected line and the control line.

### ST infection

All experiments with chickens were performed under the guidance of ethical regulation from the Institute of Animal Science, Chinese Academy of Agricultural Sciences, Beijing, China. To compare the resistance of different H/L groups to *Salmonella*, we conducted *Salmonella typhimurium* infection experiments in individuals from different generations. Specifically, selection line and non-selection line chicks from generation 10^th^, 12^th^ and F3 groups having high and low H/L values were obtained from the Changping Experimental Base of Institute of Animal Sciences (Beijing, China), while SPF white leghorn chicks were sourced from a commercial breeder (Boehringer Ingelheim Vital Biotechnology Co. Ltd, Beijing, China). The test design is detailed in Supplementary Notes 1–3. Selected chicks were raised in separate cages at the experimental center of China Agricultural University (Beijing, China) with free access to feed and water. *Salmonella typhimurium* (ST, 21484 standard strain) was purchased from the China Industrial Microbial Culture Preservation Center (Beijing, China). The bacteria were resuscitated overnight in Luria-Bertani broth (Amresco, Washington, DC) at 37 °C in an orbital shaking incubator at 150 rpm/min. After recovery, bacteria were cultured for 12 h, concentrated by centrifugation, and plated into serial dilutions from which the final number of colony-forming units (CFUs) was determined. At seven or ten days of age, the chicks were orally inoculated with 1 mL culture containing more than 2.5 × 10^10^ CFUs^56,57^ of *Salmonella typhimurium*. The mortality rate, blood samples, heterophils, liver, spleen, cecum, and cecal tonsils were collected after infection (18 or 72 h postinfection).

### Isolation of peripheral blood heterophils

Heterophils were isolated from the peripheral blood of chicks at eight days post-hatching. Blood from chicks was collected in vacutainer tubes containing disodium ethylenediaminetetraacetic acid (EDTA) (BD vacutainer, Franklin Lakes, NJ) and mixed thoroughly. The collected blood for each chick was diluted 1:1 with RPMI-1640 media containing 1% methylcellulose and centrifuged at 50 *g* for 30 min. The supernatant was then transferred to a new conical tube and diluted with Ca^2+^- and Mg^2+^-free Hank’s balanced salt solution (1:1), layered onto discontinuous Histopaque® gradients (specific gravity 1.077 over 1.119) and centrifuged at 800 g for 30 min. The heterophil layers were subsequently collected, washed with RPMI-1640 (1:1), and pelleted by centrifugation at 500 g for 5 min. The cells were then re-suspended in fresh RPMI-1640, counted on a haemocytometer, and diluted to 1 × 10^7^ /ml in RPMI-1640. All tissue culture reagents and chemicals including endotoxin-free RPMI-1640, Hank’s balanced salt solution, methylcellulose, Histopaque® 1119, and Histopaque® 1077 were obtained from Sigma Chemical Company (St. Louis, MO).

### Whole-genome resequencing

Individuals from 5th generation, 9th generation, and non-selected chicken breeds were collected to identify regions of the genome that were likely to have been targeted during periods of domestication and improvement. All chickens were obtained from the Changping Experimental Base of the Institute of Animal Sciences (Beijing, China). We selected 227 individuals for resequencing (5th generation, *n* = 43; 9th generation, *n* = 92; nonselection line, *n* = 92), along with 141 individuals from the F2 segregating population. We constructed the chicken F2 population from 2016 onward at the chicken farm of the Institute of Animal Science, Chinese Academy of Agricultural Sciences. For all birds, blood was obtained from wing veins and rapidly frozen and held at −20 °C. Total genomic DNA was extracted using a traditional phenol-chloroform protocol, and the quality and quantity of DNA were examined using a NanoDrop device and agarose gel electrophoresis.

After ensuring sample quality, paired-end libraries were generated for each eligible sample using standard procedures. The average insert size was 500 bp, and the average read length was 150 bp. All libraries were sequenced on an Illumina® HiSeq X Ten (natural populations) or HiSeq 4000 (F2 animals) (San Diego, CA) to an average raw read sequence coverage of 10×. This depth ensured the accuracy of variant calling and genotyping and met the requirements for population genetic analysis.

### Variant discovery and genotyping

Raw data were processed with Perl scripts to ensure the use of quality data in further analyses. The filtering criteria were as follows: (1) remove reads containing adapter sequence (more than five bases); (2) remove low-quality reads (more than 50% of bases having Phred Quality value less than 19); and (3) remove reads in which more than 5% of bases are N. In paired-end sequencing data, both reads of a pair would be filtered out if either was adaptor-polluted. The filtered data were evaluated in terms of quantity and quality, including Q30, data quantity, and base content statistics. The Burrows-Wheeler aligner^58^ was used to map clean reads to the chicken reference genome (ftp://ftp.ensembl.org/pub/release-96/fasta/gallus_gallus/dna/). Samtools v1.2^59^ was used to sort reads, and Mark Duplicates in Picardtools v1.13 (http://broadinstitute.github.io/picard/) to remove duplicate reads resulting from PCR. Reads mapped to two or more places were likewise filtered out. Statistics were tabulated with our in-house Perl script. The Genome Analysis Toolkit^60^ Haplotype Caller was used for SNP calling via local re-assembly of haplotypes for the population. SNPs were then filtered before further analysis using the GATK Variant Filtration tool with the following settings: QD <2.0, ReadPosRankSum < −8.0, FS > 60.0, QUAL <30.0, DP < 4.0.

### Principal component analysis (PCA)

Principal component analysis (PCA) was performed on all SNPs (chr1-chr28) using Plink (version 1.9) for the analysis of population structure. Generation 5, generation 9, and non-selection populations were separated by the first two principal components. Figures using the first and second principal components were plotted with R packages.

### Structural analysis

We used the genome-wide unlinked SNP data set and the model-based assignment program ADMIXTURE 1.3.0 to quantify genome-wide admixture between generation 5, generation 9, and nonselection populations, thereby estimating the ancestry of each individual. To estimate the parameter standard errors used to determine the optimal group number (K), ADMIXTURE was run with 200 bootstrap replicates for each possible group number (K = 2 to 7).

### Effective population size (Ne) estimation based on genomic data

In this study, linkage disequilibrium (LD) pruning was then conducted with a window size of 25 SNPs, a step of five SNPs, and r2 threshold of 0.2, yielding 640,054 independent SNP markers and LD blocks through PLINK software v1.90. After that, the SNeP software v1.1 was used to estimate the effective population size of each generation. the formula:

1 $${N}_{T(t)}=\frac{1}{\left(4{{{{{\rm{f}}}}}}\left({{{{{{\rm{c}}}}}}}_{t}\right)\right)}\left(\frac{1}{E\left[{r}_{{adj}}^{2}{{{{{\rm{|}}}}}}{c}_{t}\right]}-\alpha \right)$$

${N}_{T(t)}$ means the estimated effective population size before the past *t* generations, ${{{{{{\rm{c}}}}}}}_{t}$ means the recombination rate before the past *t* generations, ${r}_{{adj}}^{2}$ means the estimate of linkage disequilibrium, corrected for sample bias.

### Genome scanning for divergent regions

We detected candidate divergent regions (CDRs) by searching the genome for regions having high fixation index (Fst, top 1%) values and high differences in genetic diversity (Pi ratio). First, we calculated the Fst and Pi ratio along the autosomes in sliding 40-kb windows with 10-kb steps using VCF tools and in-house scripts, comparing values between generation 9 and the non-selection breeds. We restricted our CDR descriptions to the top 1% most significant windows in both Fst and ln Pi ratio, as these windows represented the extreme ends of the distributions.

The differences in allele frequencies between the two populations observed here could be driven by genetic drift and selection. To unravel these two processes, we developed a statistical test based on the assumption that genetic drift affects the whole genome, while selection affects only SNPs that are in LD with causal genes. Allele frequencies were used as test statistics. For each SNP, we tested the null hypothesis that Fst was driven purely by genetic drift against the alternative hypothesis that it was driven by both genetic drift and selection. In this process, we simulated the effect of genetic drift stochastically, which we were able to do because, as described above, the breeding history of each line from their common base population is known. In the first ten rounds of selection, 30 males and 90 females were selected for each line, which resulted in an effective population size (N_e_) of 90. To verify this number, we calculated N_e_ using the genomic data of the 9th generation and the NS population, which yielded an effective population size for the base population of approximately 200 (Supplementary Data 3). To be cautious, we used 90 as the N_e_ value in subsequent analysis.

SNPs in the significantly selected signal window were extracted and filtered based on LD and individual SNP Fst values, and the MAF values of related SNPs in the G9 and NS populations were calculated. Based on the N_e_ and MAF values of the NS population, genetic drift was simulated over the course of nine generations, and the allele frequencies at the end of the simulation were used as indicators; specifically, the means of the top 5% and the bottom 5% were calculated and compared with the allele frequencies of the G9 and NS populations.

### Genome-wide association study (GWAS)

Briefly, we started by filtering out SNPs with an inheritance or genotyping error, minor allele frequency <5%, or call rate <95%; this left a total of 8,788,385 SNPs and 249 individuals (5th generation, *n* = 35; 9th generation, *n* = 73; F2 population, *n* = 141) for analysis. Linkage disequilibrium (LD) pruning was then conducted with a window size of 25 SNPs, a step of five SNPs, and r2 threshold of 0.2, yielding 640,054 independent SNP markers and LD blocks. Principal component analysis was conducted using eigenvalues as coordinates to visualize the sample structure. The Efficient Mixed-Model Association eXpedited (EMMAX) tool was then applied to all informative SNPs with the kinship matrix (Balding-Nichols) for genome-wide association^61^. EMMAX is efficient for controlling population stratification, especially the between-generation genotype differences central to the present study^61,62^; considering population structure helps minimize false positives and increases statistical power. After this analysis, a Manhattan plot was constructed from the calculated -log_10_ (*P*-value) for each SNP. The threshold for genome-wide significance was determined based on 5% Bonferroni correction with the estimated 640,054 independent markers, giving a value of 0.05/640,054 = 7.81 × 10^−8^ [−log_10_ (*P* value) = 7.1], while the threshold for suggestive significance was 1/640,054 = 1.56 × 10^−6^ [−log_10_(*P*-value) = 5.8]. The genomic inflation factor was calculated by the GenABEL R package^63^.

### Transcriptome sequencing and analysis

Total RNA from multiple tissues (liver, spleen, cecum and cecal tonsils) collected during the ST infection experiment was isolated with QIAGEN kits (Qiagen, Hilden, Germany) and then purified for RNA-seq library construction. After all RNA libraries were produced, they were sequenced on the HiSeq X Ten platform (Illumina) using the 150 bp paired-end sequencing module. The average output was 6 Gb per library. The chicken reference genome was 6.0 version (ftp://ftp.ensembl.org/pub/release-96/fasta/gallus_gallus/dna/). Bowtie2 v2.2.3 was used to build the genome index and filtered reads. Then clean data were aligned to the reference genome using HISAT2 v2.1.0. The reads count of gene was determined by HTSeq v0.6.0, and the fragments per kilobase million mapped reads (FPKM) were calculated to estimate gene expression in each sample. Differential gene expression analysis was conducted using DESeq2 v1.6.3, which estimated the expression level of each gene per sample by linear regression, then calculated the associated *P*-value using the Wald test. Those genes with fold-change ≥ 2 and *P* < 0.05 were considered to be differentially expressed genes (DEGs).

Heterophils were collected from the F3 population (6 chicks with three low H/L and three high H/L individuals) and Selection line 12^th^ generation (16 chicks with 8 high *PTPRJ* expression and 8 low *PTPRJ* expression) after ST infection using a single-cell collection solution that contained cell lysis components and RNase inhibitors. Reverse transcription was performed with oligo(dT) to form first-strand cDNA. PCR amplification was then used to enrich cDNA, and the amplified product was purified before library construction, which included the steps of DNA fragmentation, end repair, adding “A” plus linker, PCR amplification and library quality control. The constructed library was then sequenced as 150-bp paired-end (PE) reads using the Illumina platform. In order to guarantee data quality, a Perl script was applied to filter the original reads (Raw Data). The script employed the following steps: 1) Trim Smart-seq2 public primer sequence from reads (trimmed reads with length less than 30 bp were discarded); 2) Remove reads contaminated by adapters (reads were defined as contaminated if they contained more than 5 bp of adapter sequence); 3) Remove low-quality reads (reads were defined as low-quality if more than 15% of bases had phred quality value less than or equal to 19); 4) Remove reads in which N bases comprise more than 5% of the total bases. At each step, if one read in a pair was discarded, the other was also too. The clean data were aligned to the reference genome using HISAT2 v2.1.0. The reads count of gene was determined by HTSeq v0.6.0, and the fragments per kilobase million mapped reads (FPKM) were calculated to estimate gene expression. Analysis to identify DEGs was conducted using DESeq2. Genes with *P* < 0.05 and |log2_ratio| ≥ 1 (Selection line 12^th^ generation) or |log2_ratio| ≥ 1 and *Padj* < 0.05 (F3 population) were identified as DEGs.

### Construction of reporter plasmids and dual-luciferase reporter assays

Informed by the multiple cloning site of the pGL4.18 vector, primers amplifying two target regions associated with *PTPRJ* were designed as follows: the XhoI restriction site was included in the 5’ primer and the HindIII restriction site in the 3’ primer, and the amplified regions consisted of the promoter upstream of the transcription start site (-1500-+1) and an about 600-bp area located downstream of the gene (downstream, ds; includes a SNP site). For the downstream region, DNA fragments containing rs736799474 [C] and rs736799474 [T] were amplified by PCR from the corresponding homozygous DNA samples. Primer sequences were: LIC fwd primer for chicken *PTPRJ* promoter with XhoI site: CCTGAGCTCGCTAGCCTCGAGctgtcaggtattggatatagg; LIC rev primer for chicken *PTPRJ* promoter with HindIII site: CAGTACCGGATTGCCAAGCTT agcagcggcagccgcctcat; LIC fwd primer for chicken *PTPRJ* downstream with XhoI site: CCTGAGCTCGCTAGCCTCGAGagcaaatgtctcttatcct; LIC rev primer for chicken *PTPRJ* downstream with HindIII site: CAGTACCGGATTGCCAAGCTTcgcatgtaacactgtaacat; fwd primer for mutation from C to T: GAAATGCTAAGTCACAGtTTGAAGCAGTGATTG AGCACCTGGTGGGAA; rev primer for mutation from C to T: AATCACTGCTTCAAaCTG TGACTTAGCATTTCCACTACACAAGACCAG. The obtained PCR products were then cloned into the pGL4.18 firefly luciferase expression vector to generate *PTPRJ* promoter-reporter plasmids. Dual-luciferase reporter assays were carried out using DF_1_ cells in passage ten that had been maintained in DMEM medium with 10% FBS in humidified 5% CO_2_ at 37 °C. All cells were free of mycoplasma infection. For the assays, 5 × 10^4^ DF_1_ cells were seeded in 48-well plates, then transfected with the appropriate reporter constructs and with the pBEC22 control vector *Renilla* luciferase for normalization of luciferase activity. Luciferase activity was measured at 48 hours using the Dual-Luciferase Reporter System (Promega, Madison, WI). For each plasmid construct, four independent transfection experiments were performed in triplicate.

### Electro*p*horetic mobility shift assays (EMSAs)

Nuclear proteins from heterophils were extracted with a protein extraction kit according to the kit’s instructions (Thermo Fisher Scientific, Waltham, USA). Oligonucleotides used in the EMSA were as follows: *PTPRJ*-probe-WT-Bio: GGAAATGCTAAGTCACAGCTTGAAGCAGTGATTGAGC; *PTPRJ*-probe-Mut-Bio: GGAAATGCTAAGTCACAGTTTGAAGCAGTGATTGAGC. Five microliters of nuclear proteins (5 μg/μl) were incubated with 10 pmol of biotin-labeled probes containing either the C or T allele. Simultaneously, cold competition controls were incorporated by adding 200-fold excess of unlabeled competitors containing the C or T allele. Finally, a picture was obtained using an EMSA kit (Thermo Fisher Scientific, Waltham, USA).

### Genotyping by MassARRAY and genetic correlation analysis

Genomic DNA was extracted from Chinese-native Jin Ling Hua (JLH) chickens (*n* = 384), and its quality and quantity were determined using a NanoDrop spectrometer and agarose gel electrophoresis analysis. MassARRAY detection was performed by Beijing Compass Biotechnology Co., Ltd. (Beijing, China) using the MassARRAY® analyzer (Agena Bioscience, San Diego, CA) to characterize the genotypes of candidate SNPs. Briefly, specific amplification of candidate SNPs was performed in a Veriti® 384-Well Thermal Cycler (Applied Biosystems, Foster City, CA) and the PCR products treated with alkaline phosphatase. A single-base extension reaction and resin purification were carried out, after which the PCR products were hybridized to 384 chips for mass spectrometry detection. Finally, single SNP correlation analysis of 21 SNPs in a total of 384 individuals was performed using a general linear model with PLINK.

### Statistics and Reproducibility

The significance of differences between groups was tested using the paired-sample or Student’s *t*-test in SPSS version 22.0 (IBM Corp., Armonk, NY). Confidence limits were set at 95%, *P* < 0.05 (*) or *P* < 0.01 (**) were considered significantly different. Data were expressed as the mean ± standard deviation (SD) unless otherwise indicated. Box plots of SNP combinations based on H/L phenotype were produced with the R package *ggpubr*. The range of sample sizes used in this research was shown in Table 2.

**Table 2:** Range of sample sizes used in this research.

Experiment	Breeds	Group	Number
ST infection	SPF White leghorn	low H/L	28
		high H/L	19
	JXH chicken	low H/L	72
		high H/L	70
	JXH chicken	low H/L	40
		middle H/L	45
		high H/L	45
	10th generation	selected	35
		non-selected	60
	10th generation	selected	116
		non-selected	30
	12th generation	selected	90
		non-selected	90
MassARRAY	JLH chickens	\	384
Whole-genome resequencing	5th generation	selected	43
	9th generation	selected	92
	non-selected line	non-selected	92
	F2 population	\	141
Transcriptome sequencing	liver, spleen, cecum and cecal tonsils (JXH chicken)	selected	8
		non-selected	8
	Heterophils (F2 population)	low H/L	3
		high H/L	3
	Heterophils (12th population)	low H/L	8
		high H/L	8
Dual-luciferase reporter assays	DF1	\	4 replicates per vector

A conventional pedigree-based best linear unbiased prediction model (BLUP) was used to predict breeding values for the JLH H/L ratio. The pedigree-based BLUP model^64^ is

2 $$y={{{{{\rm{xb}}}}}}+{{{{{\rm{Za}}}}}}+{{{{{\rm{e}}}}}}$$

where *y* is the vector of the phenotypic records of the trait (H/L), b is the vector of fixed effects (batch and sex), x is the incidence matrix linking b to *y*, a is the vector of additive breeding values to be estimated, Z is the incidence matrix linking a to *y*, and e is the vector of residuals. We assumed that ${{{{{\rm{var}}}}}}({{{{{\rm{a}}}}}})={{{{{\rm{A}}}}}}{\sigma }_{a}^{2}$, where A is the pedigree-based genetic relationship matrix. The estimated breeding value (EBV) was determined with the Asreml package^65^ and used for the next SNP analysis. The heritability of the H/L ratio based on the H/L selection line was also estimated with the same pedigree-based BLUP model.

### Reporting summary

Further information on research design is available in the Nature Portfolio Reporting Summary linked to this article.

## Supplementary information

Supplementary Information

Description of Additional Supplementary Files

Supplementary Data 1-11

Supplementary Data 12

Reporting Summary

## Data Availability

The sequencing data reported in this research are available from National Genomics Data Center under the BioProject no. PRJCA004071, PRJCA004075 and PRJCA014424 (https://ngdc.cncb.ac.cn). All source data underlying the graphs presented in the main figures were uploaded as Supplementary Data 12.
