# Supplementary material for: A heterophil/lymphocyte-selected population reveals the phosphatase PTPRJ is associated with immune defense in chickens
Source: Commun Biol. 2023 Feb 18;6:196. doi: 10.1038/s42003-023-04559-x (PMC9938895; doi:10.1038/s42003-023-04559-x)

Supplementary Material

Supplementary Notes

Salmonella infection

Supplementary Note 1: *Salmonella typhimurium* infection in Generation 10

In generation 10, selection line and non-selection line chicks were obtained from the Changping Experimental Base of Institute of Animal Sciences (Beijing, China). A total of 146 one-day-old chicks (Table 1) were raised in separate cages at the experimental center of China Agricultural University (Beijing, China) with free access to feed and water. *Salmonella typhimurium* (ST, 21484 standard strain) was purchased from China Industrial Microbial Culture Preservation Center (Beijing, China). The bacteria were resuscitated overnight in Luria–Bertani (LB) broth (Amresco, Washington, DC, USA) at 37 °C in an orbital shaking incubator at 150 rpm. After recovery, the bacteria were cultured for 12 h and concentrated in a centrifuge. The final number of colony forming units (CFUs) was determined by plating serial dilutions. At seven days of age, the chicks were orally inoculated with 1 mL culture containing 2.5×10^{10} CFU *Salmonella typhimurium*. Blood samples, livers, spleen, cecum, and cecal tonsils were collected at three days post infection.

Supplementary Note 2: *Salmonella typhimurium* infection in Generation 12

In generation 12, selection line and non-selection line chicks were obtained from the Changping Experimental Base of Institute of Animal Sciences (Beijing, China). A total of 180 one-day-old chicks were raised in separate cages at the experimental center of China Agricultural University (Beijing, China) with free access to feed and water. *Salmonella typhimurium* (ST, 21484 standard strain) was purchased from China Industrial Microbial Culture Preservation Center (Beijing, China). The bacteria were resuscitated overnight in Luria–Bertani (LB) broth (Amresco, Washington, DC, USA) at 37 °C in an orbital shaking incubator at 150 rpm. After recovery, the bacteria were cultured for 12 h and concentrated in a centrifuge. The final number of colony forming units (CFUs) was determined by plating serial dilutions. At seven days of age, the chicks were orally inoculated with 1 mL culture containing 8.97×10^{11} CFU *Salmonella typhimurium*. The mortality rate and heterophil and leukocyte counts in peripheral blood were determined at 24 hours days post infection, Counts were compared with non-infected chicks and between selection and non-selection lines.

Supplementary Note 3: *Salmonella typhimurium* infection in F2 population

In the F2 population, extreme individuals among the high and low H/L groups were selected and mated to two F3 populations (high H/L group and low H/L group). A total of 60 one-day-old chicks from these crosses were raised in separate cages at the experimental center of China Agricultural University (Beijing, China) with free access to feed and water. *Salmonella typhimurium* (ST, 21484 standard strain) was purchased from China Industrial Microbial Culture Preservation Center (Beijing, China). The bacteria were resuscitated overnight in Luria–Bertani (LB) broth (Amresco, Washington, DC, USA) at 37 °C in an orbital shaking incubator at 150 rpm. After recovery, the bacteria were cultured for 12 h and concentrated in a centrifuge. The final number of colony forming units (CFUs) was determined by plating serial dilutions. At seven days of age, the chicks were orally inoculated with 1 mL culture containing 5.27×10^{10} CFU *Salmonella typhimurium*. The mortality rate and heterophils in blood were determined at 24 hours days post infection.

Supplementary Figures

Supplementary Figure 1. Diagram of F3 population intercross between the selection and non-selection lines.

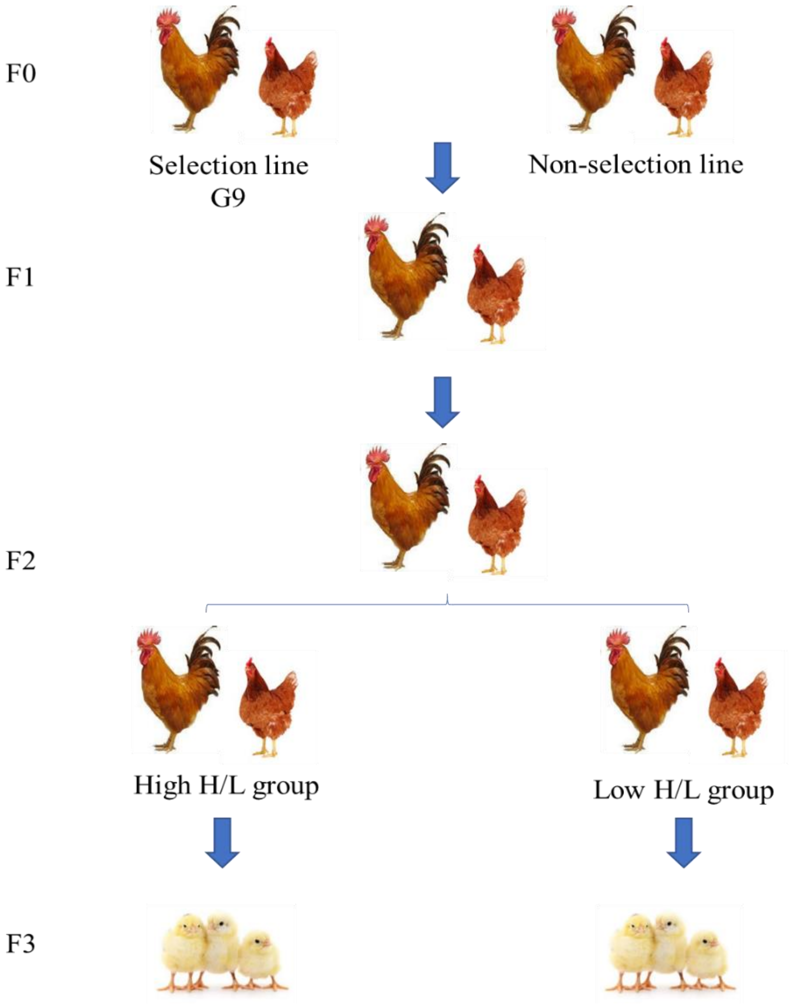

Supplementary Figure 2. Principal component analysis.

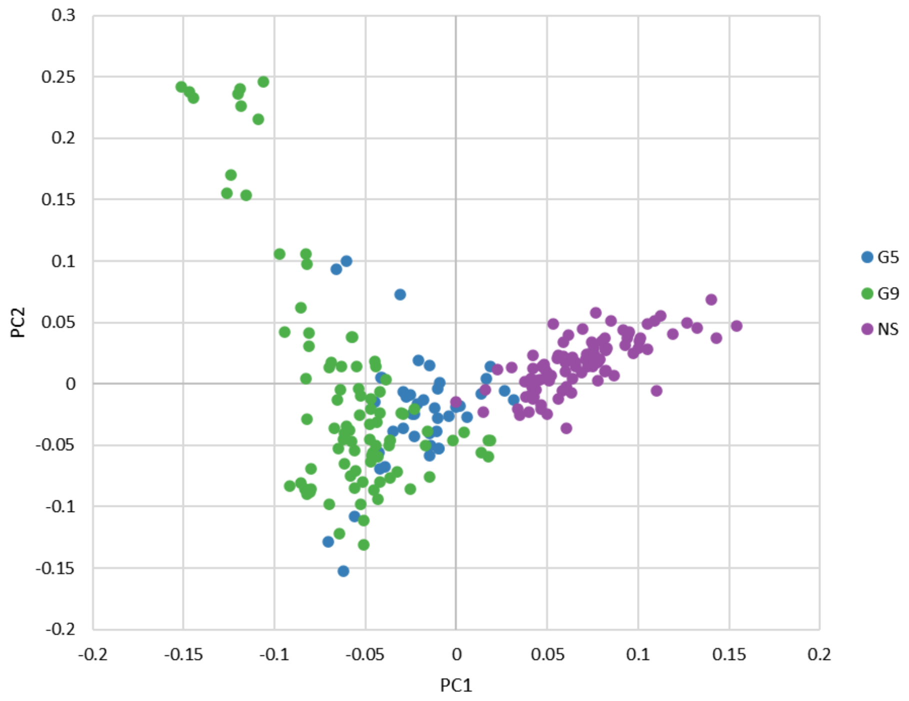

Supplementary Figure 3. Genome-wide admixture analyses inferred from SNPs analyzed with ADMIXTURE 1.3.

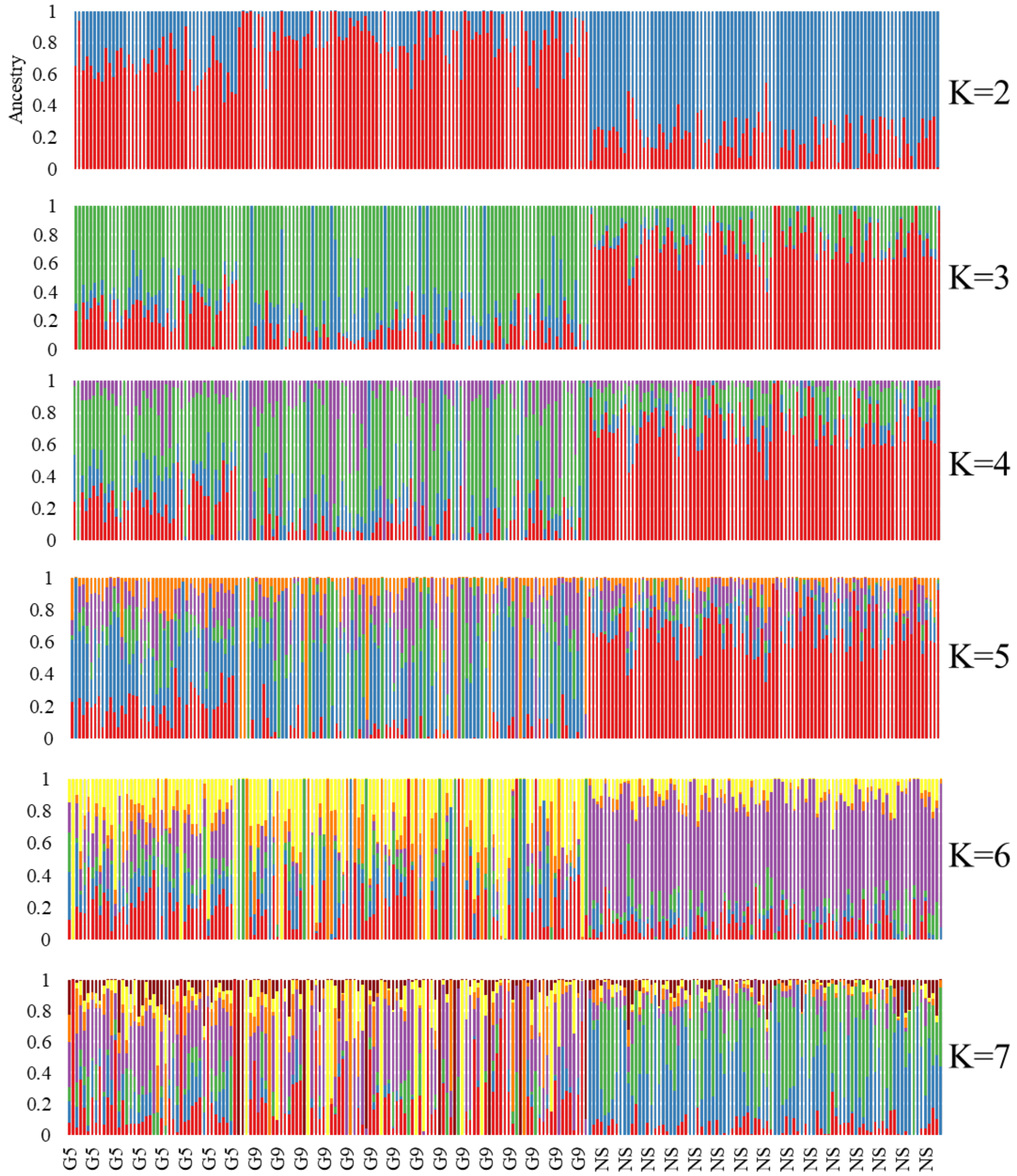

At K = 2, 3 and 4, the three populations (non-selection line, 5th generation, and 9th generation) separate into three groups, which indicates a potential slight gene differentiation between the selection and non-selection lines.

Supplementary Figure 4. Genome-wide distribution of the pi ratio and Fst, determined across all autosomes using 40-kb windows with 10-kb steps.

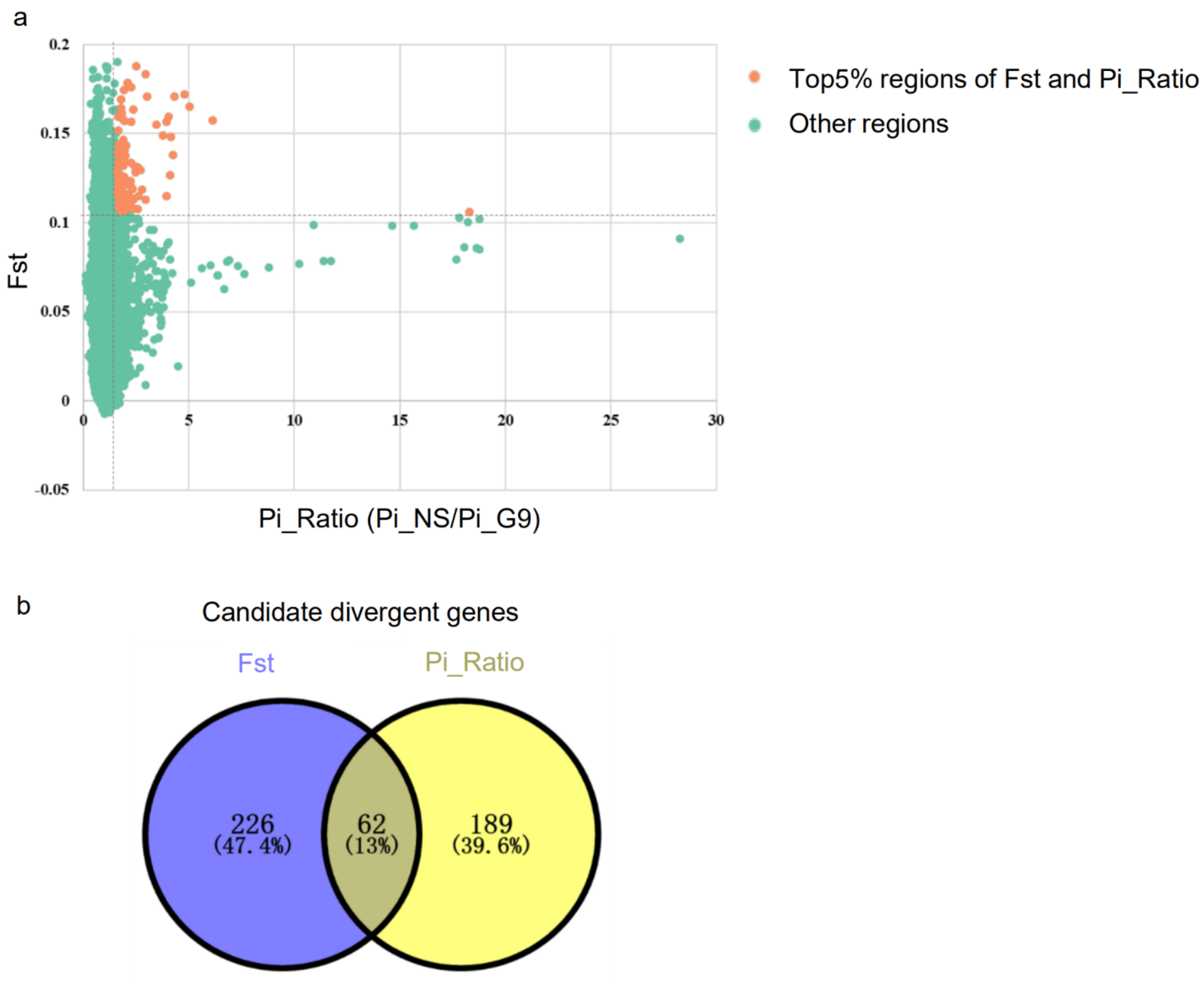

a Comparison of generation 9 and non-selection breeds. Orange dots represent windows fulfilling the requirement for selected regions. The gray vertical dashed line indicates the pi ratio threshold, and the gray horizontal dashed line the Fst threshold, pi ratio=(pi_G9/pi_NS). **b** Intersection of the candidate divergent gene sets identified by Fst and pi ratio.

Supplementary Figure 5. Differential gene expression between selection and non-selection lines or high and low H/L groups in multiple tissues.

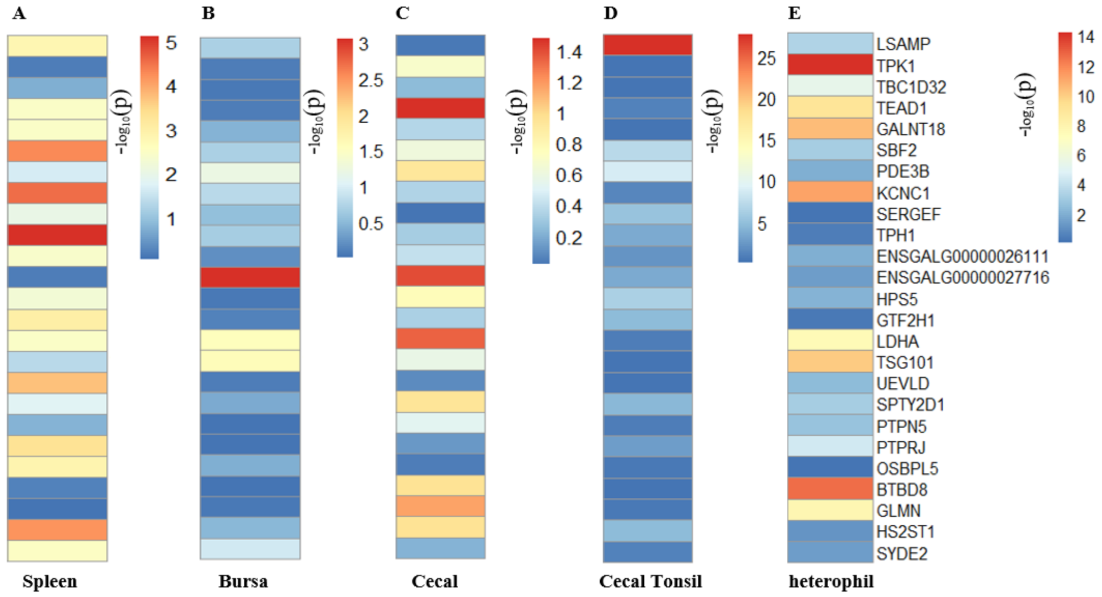

a Spleen, **b** bursa, **c** cecum, **d** cecal tonsils, and **e** heterophils. *P*-values are for the comparison of selection line vs. non-selection line chickens (for heterophils, *P*-values compare the high H/L group vs low H/L group) based on RNA-seq data and obtained using Fisher's exact test via Deseq2. Values shown are -log (*P*).

Supplementary Figure 6. Trend plot of allele frequency changes.

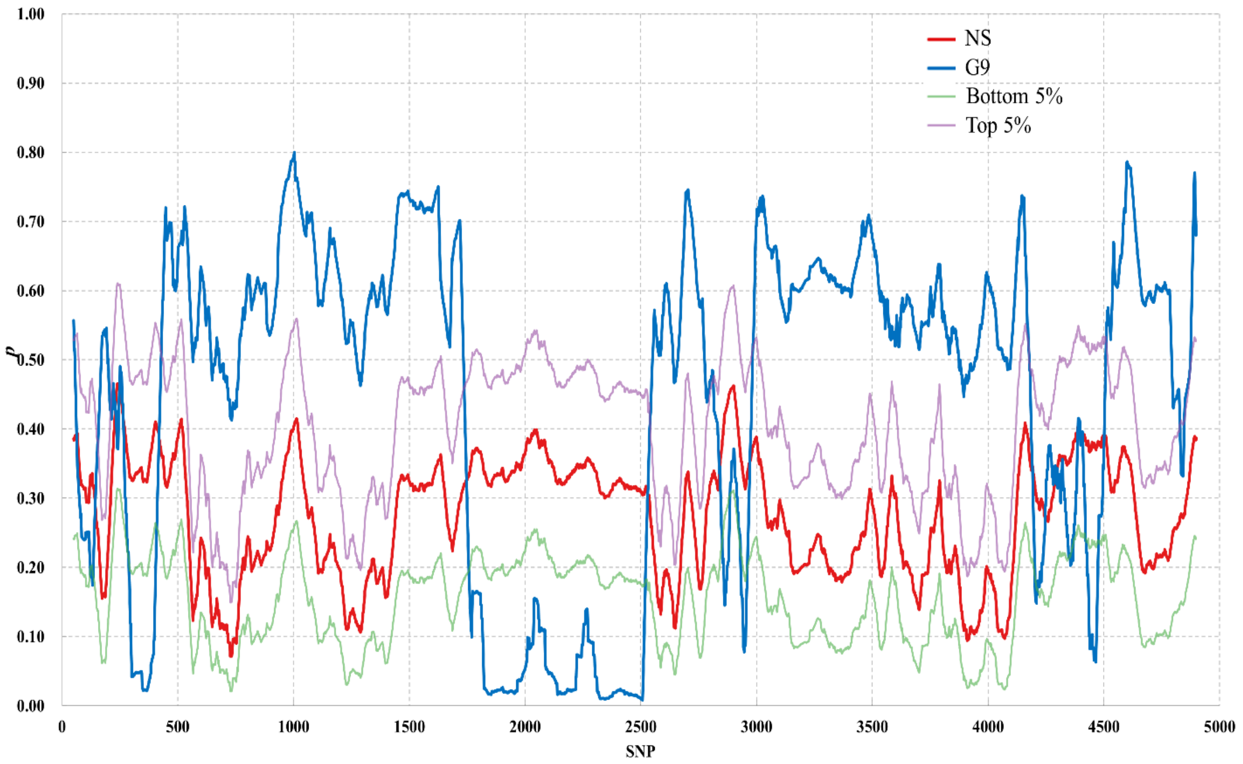

The red line shows the NS population allele frequencies, green and purple show the bottom 5% and top 5% allele frequencies based on the NS population simulation, respectively, and blue line shows the G9 population allele frequencies.

Supplementary Figure 7. Gene expression of *PTPRJ*, *LYN*, *SHP-1* and *SHIP-1* in individuals from the F3 population with high and low H/L. Data are expressed as the mean ± standard deviation (SD).

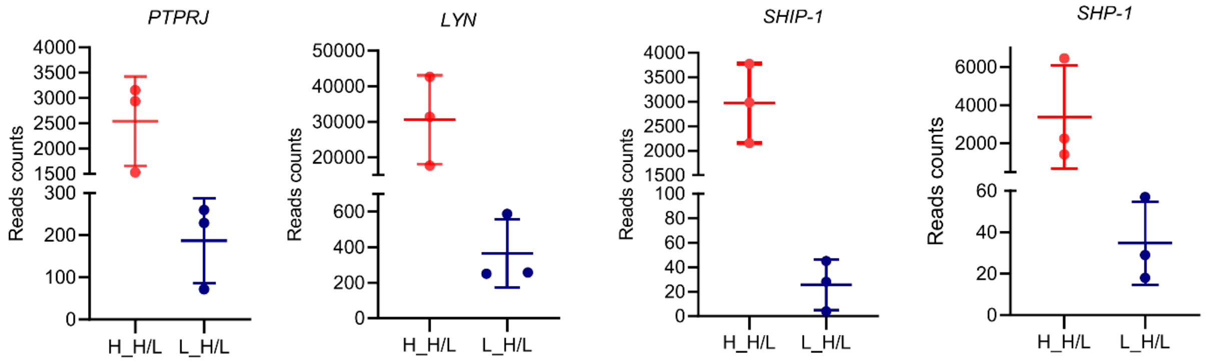

Supplementary Figure 8. *PTPRJ* exerts an inhibitory effect on the Toll-like receptor and Fcγ Rs signaling pathways through the *LYN* gene.

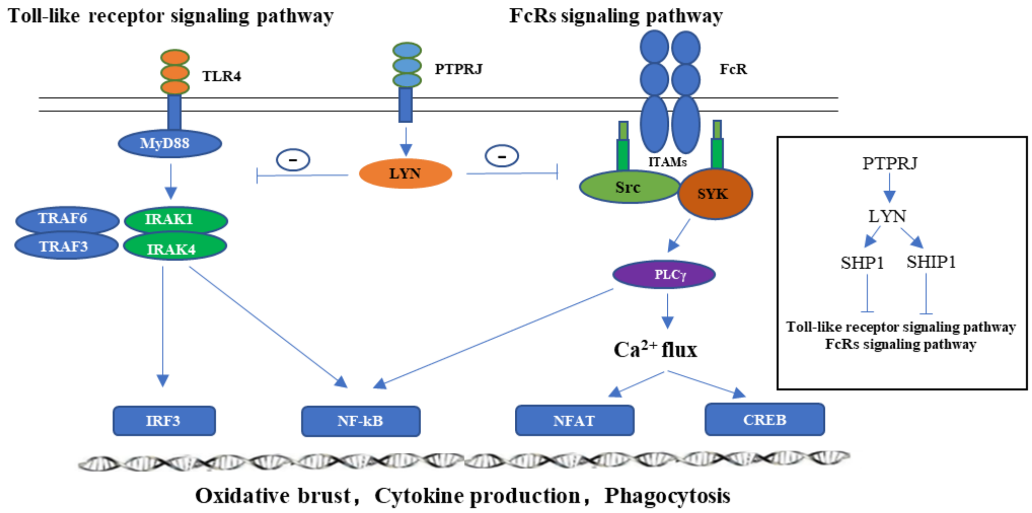

Bacterial factors such as lipopolysaccharides (LPS) activate innate immunity as well as stimulate the antigen-specific immune response and trigger the inflammatory response. Members of the Toll-like receptor (TLR) gene family convey signals stimulated by these factors, activating signal transduction pathways that result in transcriptional regulation and stimulate immune function. TLR4 is activated by

LPS. The downstream signaling pathways used by these receptors activate IL-1 receptor associated kinase (IRAK) through the MyD88 adaptor protein, and subsequent signaling through TRAF-6 and protein kinase cascades activates NF-kB. NF-kB then activates transcription of genes such as those encoding the proinflammatory cytokines IL-1 and IL-12. When Fcγ receptors are involved in binding, the γ chain of FcγR and the ITAM tyrosine of FcγRIIA are rapidly phosphorylated. The two Src homology domains in the tyrosine kinase Syk bind to ITAM, leading to formation of signal complexes on the cell membrane. Within those complexes, Syk-mediated phosphorylation of effector proteins activates downstream signaling pathways that can culminate in cell phagocytosis, degranulation, and cytokine and peroxide release.

Supplementary Figure 9. Mutation function prediction for rs736799474

a

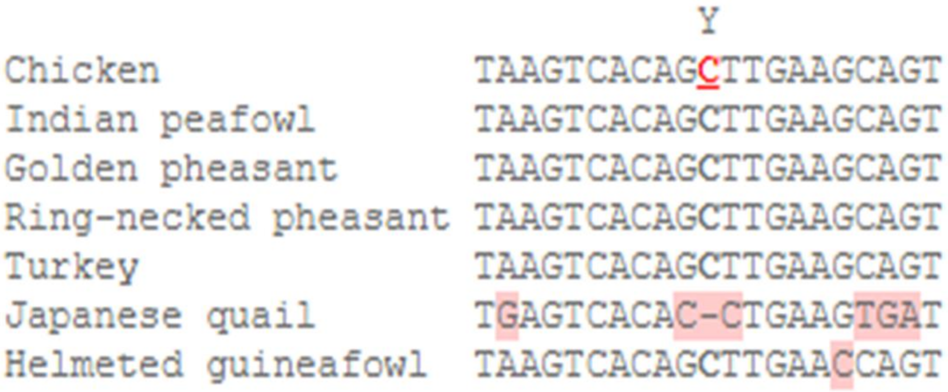

b

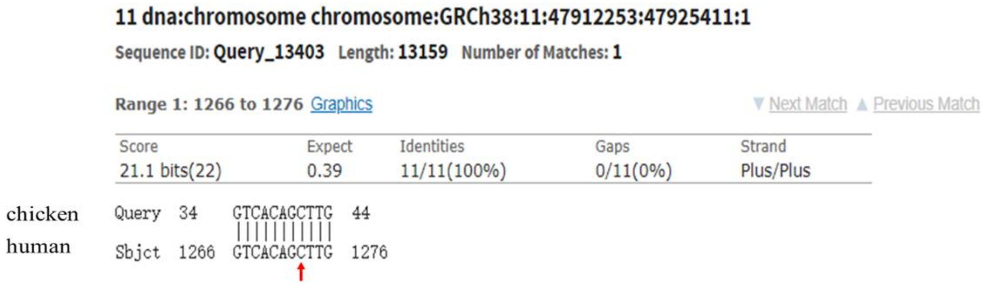

According to the reference genome annotation, rs736799474 is located downstream of the *PTPRJ* gene. **a** Multi-species sequence alignment indicates that the region containing it is conserved. **b** According to the Genecard entry for *PTPRJ* (https://www.genecards.org/cgi-bin/carddisp.pl?gene=PTPRJ&keywords=PTPRJ), the human *PTPRJ* gene features a132-Kb enhancer region located 61.7 Kb downstream. Alignment of this region with the sequence around the chicken SNP revealed that a 10-bp sequence including the rs736799474 mutation site is highly conserved. Therefore, we speculate that the sequence near rs736799474 has enhancer activity.

Supplementary Figure 10. Unprocessed scans of western blots.

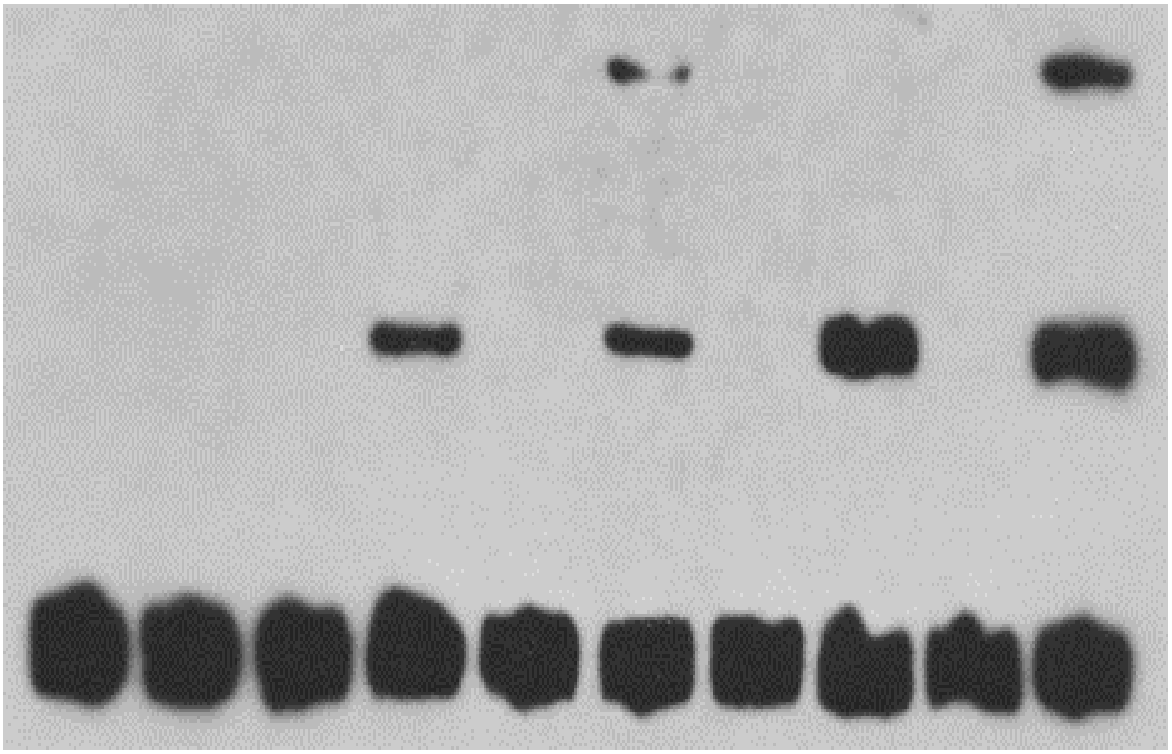

Supplement: Supplementary file 1 — Supplementary Information [file 42003_2023_4559_MOESM1_ESM.pdf]
