# Supplementary material for: A heterophil/lymphocyte-selected population reveals the phosphatase PTPRJ is associated with immune defense in chickens
Source: Commun Biol. 2023 Feb 18;6:196. doi: 10.1038/s42003-023-04559-x (PMC9938895; doi:10.1038/s42003-023-04559-x)
Supplement: Supplementary file 2 — Description of Additional Supplementary Files [file 42003_2023_4559_MOESM2_ESM.docx]

**Description of Additional Supplementary Files**

**File name:**Supplementary Data 1

**Description:**Mortality of different groups after infection with ST.

**File name:**Supplementary Data 2

**Description:** Statistic parameters of population diversity in three chicken groups.

**File name:**Supplementary Data 3

**Description:**Effective population size (Ne) estimation based on genomic data.

**File name:**Supplementary Data 4

**Description:**H/L ratios in the F2 populations used for breeding the F3 population.

**File name:**Supplementary Data 5

**Description:**Expression of candidate divergent genes.

**File name:**Supplementary Data 6

**Description:**GWAS significant SNPs.

**File name:**Supplementary Data 7

**Description:**Annotations of GWAS significant SNPs.

**File name:**Supplementary Data 8**.**

**Description:**Selection signal in the GWAS significant region.

**File name:**Supplementary Data 9

**Description:**Mapping details of resequencing samples.

**File name:**Supplementary Data 10

**Description:**KEGG enrichment results of differentially expressed genes.

**File name:**Supplementary Data 11

**Description:**GO enrichment results of differentially expressed genes.

**File name:**Supplementary Data 12

**Description:**All source data underlying the graphs presented in the main figures.
