# Supplementary material for: A heterophil/lymphocyte-selected population reveals the phosphatase PTPRJ is associated with immune defense in chickens
Source: Commun Biol. 2023 Feb 18;6:196. doi: 10.1038/s42003-023-04559-x (PMC9938895; doi:10.1038/s42003-023-04559-x)
Supplement: Supplementary file 5 — Reporting Summary [file 42003_2023_4559_MOESM5_ESM.pdf]

nature portfolio

Corresponding author(s): Qinghe Li, Guiping Zhao,COMMSBIO-22-0611A

Last updated by author(s): Jan 15, 2023

Reporting Summary

Nature Portfolio wishes to improve the reproducibility of the work that we publish. This form provides structure for consistency and transparency in reporting. For further information on Nature Portfolio policies, see our Editorial Policies and the Editorial Policy Checklist.

Statistics

For all statistical analyses, confirm that the following items are present in the figure legend, table legend, main text, or Methods section.

n/a	Confirmed	
☐	☒	The exact sample size (*n*) for each experimental group/condition, given as a discrete number and unit of measurement
☐	☒	A statement on whether measurements were taken from distinct samples or whether the same sample was measured repeatedly
☐	☒	The statistical test(s) used AND whether they are one- or two-sided<br>*Only common tests should be described solely by name; describe more complex techniques in the Methods section.*
☒	☐	A description of all covariates tested
☐	☒	A description of any assumptions or corrections, such as tests of normality and adjustment for multiple comparisons
☒	☐	A full description of the statistical parameters including central tendency (e.g. means) or other basic estimates (e.g. regression coefficient) AND variation (e.g. standard deviation) or associated estimates of uncertainty (e.g. confidence intervals)
☒	☐	For null hypothesis testing, the test statistic (e.g. *F*, *t*, *r*) with confidence intervals, effect sizes, degrees of freedom and *P* value noted<br>*Give P values as exact values whenever suitable.*
☒	☐	For Bayesian analysis, information on the choice of priors and Markov chain Monte Carlo settings
☒	☐	For hierarchical and complex designs, identification of the appropriate level for tests and full reporting of outcomes
☐	☒	Estimates of effect sizes (e.g. Cohen's *d*, Pearson's *r*), indicating how they were calculated

Our web collection on statistics for biologists contains articles on many of the points above.

Software and code

Policy information about availability of computer code

Data collection	All DNA libraries were sequenced on an Illumina® HiSeq X Ten (natural populations) or HiSeq 4000 (F2 animals) (San Diego, CA) to an average raw read sequence coverage of 10x.After all RNA libraries were produced, they were sequenced on the HiSeq X Ten platform (Illumina) using the 150 bp paired-end sequencing module.
Data analysis	Software we used:BWA(V0.7.17); Samtools (V1.2); Picardtools (V1.13); GATK (V4.0);Plink (V1.9); R(V 4.1.0); ADMIXTURE (V1.3.0); vcftools (V4.2); EMMAX (); TopHat (V2.1.1); SPSS (V22.0);

For manuscripts utilizing custom algorithms or software that are central to the research but not yet described in published literature, software must be made available to editors and reviewers. We strongly encourage code deposition in a community repository (e.g. GitHub). See the Nature Portfolio guidelines for submitting code & software for further information.

Data

Policy information about availability of data

All manuscripts must include a data availability statement. This statement should provide the following information, where applicable:

- Accession codes, unique identifiers, or web links for publicly available datasets
- A description of any restrictions on data availability
- For clinical datasets or third party data, please ensure that the statement adheres to our policy

The genome sequence data reported in this article is being uploaded to the genome sequence file of the BIG Data Center of the Beijing Institute of Genomics, Chinese Academy of Sciences, and is publicly available from http://bigd.big.ac.cn (CRA003684).

Field-specific reporting

Please select the one below that is the best fit for your research. If you are not sure, read the appropriate sections before making your selection.

☒ Life sciences ☐ Behavioural & social sciences ☐ Ecological, evolutionary & environmental sciences

For a reference copy of the document with all sections, see nature.com/documents/nr-reporting-summary-flat.pdf

Life sciences study design

All studies must disclose on these points even when the disclosure is negative.

Sample size	In this project, 2 chicken lines and 1 F2 population were used. These samples are representative genetic materials. The number of samples is sufficient for population genomics analysis.
Data exclusions	No exclusion of data was made.
Replication	We did three biological replications for dual-luciferase transient expression assays.<br>For sequencing data, no replication was used since it is a common practice for sequencing related experiments.
Randomization	For each experiment, chicken samples were grown in the same conditions with randomly assigned catch.
Blinding	No blinding was used since measurements were not vulnerable to observer bias.

Reporting for specific materials, systems and methods

We require information from authors about some types of materials, experimental systems and methods used in many studies. Here, indicate whether each material, system or method listed is relevant to your study. If you are not sure if a list item applies to your research, read the appropriate section before selecting a response.

Materials & experimental systems

n/a	Involved in the study	
☐	☒	Antibodies
☐	☒	Eukaryotic cell lines
☒	☐	Palaeontology and archaeology
☐	☒	Animals and other organisms
☒	☐	Human research participants
☒	☐	Clinical data
☒	☐	Dual use research of concern

Methods

n/a	Involved in the study	
☒	☐	ChIP-seq
☒	☐	Flow cytometry
☒	☐	MRI-based neuroimaging

Antibodies

Antibodies used	PTPRJ Polyclonal antibody ;Proteintech; 55123-1-AP
Validation	All antibodies used here have been validated by manufacturers. Furthermore, these antibodies have been widely used in the animal genomic studies.

Eukaryotic cell lines

Policy information about cell lines

Cell line source(s)	DF1
Authentication	None of the cell lines used were authenticated
Mycoplasma contamination	The cell lines were not tested for mycoplasma contamination
Commonly misidentified lines<br>(See ICLAC register)	None

Animals and other organisms

Policy information about studies involving animals; ARRIVE guidelines recommended for reporting animal research

Laboratory animals	chicken
Wild animals	The study did not involve wild animals
Field-collected samples	The study did not involve samples collected from the field
Ethics oversight	All animals and experimental protocols used in this study were approved by the Beijing Institute of Animal Science, Chinese Academy of Agricultural Sciences (the scientific research department responsible for animal welfare issues) (No.: IASCAAS-AE20140615).

Note that full information on the approval of the study protocol must also be provided in the manuscript.
